# Compensatory Flux Changes within an Endocytic Trafficking Network Maintain Thermal Robustness of Notch Signaling

**DOI:** 10.1016/j.cell.2014.03.050

**Published:** 2014-05-22

**Authors:** Hideyuki Shimizu, Simon A. Woodcock, Marian B. Wilkin, Barbora Trubenová, Nicholas A.M. Monk, Martin Baron

**Affiliations:** 1 University of Manchester, Faculty of Life Sciences, Michael Smith Building, Oxford Road, Manchester M13 9PT, UK; 2 School of Mathematics and Statistics, University of Sheffield, Hicks Building, Hounsfield Road, Sheffield S3 7RH, UK

## Abstract

Developmental signaling is remarkably robust to environmental variation, including temperature. For example, in ectothermic animals such as *Drosophila*, Notch signaling is maintained within functional limits across a wide temperature range. We combine experimental and computational approaches to show that temperature compensation of Notch signaling is achieved by an unexpected variety of endocytic-dependent routes to Notch activation which, when superimposed on ligand-induced activation, act as a robustness module. Thermal compensation arises through an altered balance of fluxes within competing trafficking routes, coupled with temperature-dependent ubiquitination of Notch. This flexible ensemble of trafficking routes supports Notch signaling at low temperature but can be switched to restrain Notch signaling at high temperature and thus compensates for the inherent temperature sensitivity of ligand-induced activation. The outcome is to extend the physiological range over which normal development can occur. Similar mechanisms may provide thermal robustness for other developmental signals.

## Highlights

• There are multiple routes to Notch activation, differently affected by temperature
• Dx and Su(dx) promote clathrin-dependent and -independent Notch endocytosis
• Notch endocytosis through the distinct routes can up- or downregulate signaling
• Flux changes within endocytic network ensure Notch signal is robust to temperature

## Graphical Abstract

**Figure:**
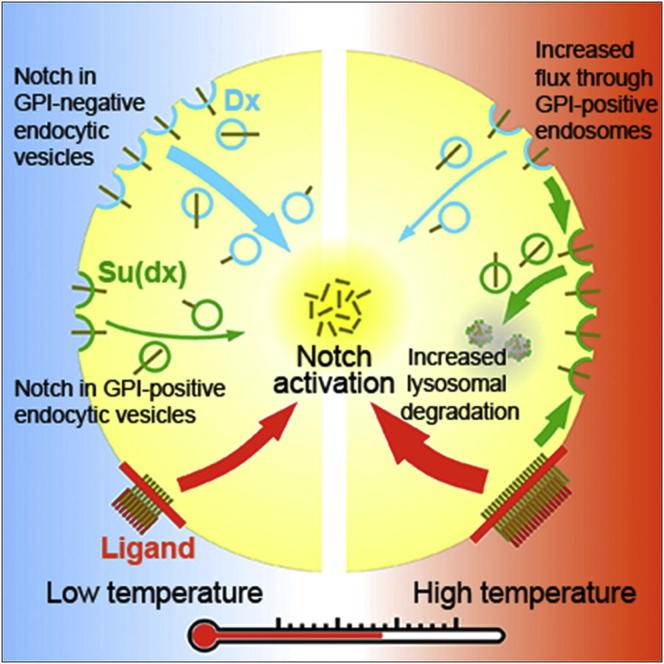

## Teaser

The robustness of Notch signaling to temperature changes during fly development is achieved by a network of competing temperature-dependent endocytic trafficking routes. These stabilize signaling levels in the face of temperature variation that influences ligand-induced receptor activation.

## Introduction

Waddington introduced the concept of canalization to describe how potential variation in development is channeled to a common endpoint (Waddington 1959; Kitano, 2004). In particular, the robustness of those ectothermic organisms such as *Drosophila* that can develop normally over a wide range of temperatures is remarkable (Lucchetta et al., 2005). Recent work has identified downstream trancriptional regulatory elements that may help to confer stable outcomes at different temperatures (Frankel et al., 2010; Li et al., 2009). However, relatively little is known regarding how temperature compensation in developmental signaling pathways preserves signaling outputs within normally tolerated thresholds. In wild-type (WT) *Drosophila,* Notch (N) receptor signaling levels are remarkably stable to temperature variation (Mazaleyrat et al., 2003). This thermal robustness is lost through mutations in genes that have been associated with N trafficking (Mazaleyrat et al., 2003; Wilkin et al., 2008). In this study, we investigate how compensatory adjustments of flux, within a network of competing endocytic trafficking routes, provides temperature insensitivity of N receptor signaling during *Drosophila* development.

N receptor signaling is utilized in many different cell fate decisions during development. N is activated by binding to membrane-bound DSL (Delta/Serrate/Lag2)-domain ligands (reviewed by Kopan and Ilagan, 2009). This results in extracellular domain cleavage by the metalloprotease Kuzbanian (Kuz) to release an intermediate membrane-bound form (NEXT), followed by Presenilin-dependent S3 cleavage in the transmembrane domain to release the N intracellular domain (NICD). NICD translocates to the nucleus, forming a transcriptional regulatory complex with Suppressor of Hairless and the coactivator protein Mastermind (Mam). Alternatively, a cytoplasmic ring finger protein Deltex (Dx) can bind and activate N independently of DSL ligands by promoting N endocytic trafficking to the lysosome (Wilkin et al., 2008; Hori et al., 2011; Baron, 2012). In this case, N cleavage and Presenilin-dependent release of NICD depends on retention of N in the lysosome limiting membrane. Mutations that prevent transfer of N from the endosomal limiting membrane into the internal endosomal compartments can thus result in considerable ectopic misactivation of N (reviewed by Fortini and Bilder, 2009). Late endosomal trafficking components such as members of the HOPS complex, including Deep orange (Dor) and Carnation (Car), are required only for the lysosomal N activation mechanisms. Their loss does not affect signals originating from ligand-induced S2 cleavage at the cell surface. Interestingly, *dx* null mutant phenotypes, which resemble N loss of function, markedly worsen with increased temperature (Wilkin et al., 2008). The *dx* mutant phenotypes are dominantly suppressed by mutations of *Suppressor of deltex* (*Su(dx)*) (Fostier et al., 1998). The latter is a HECT domain E3 ubiquitin ligase that blocks Dx-induced N activation by diverting endocytosed N from the late endosome limiting membrane into the multivesicular body (mvb), thus sequestering it from activation (Cornell et al., 1999; Wilkin et al., 2008). Thus, endocytosis of N can contribute either positively or negatively to signaling levels. The phenotype of *Su(dx)* null mutations is also temperature sensitive and, unlike WT, the mutants upregulate N signaling when shifted up to 29°C (Fostier et al., 1998; Cornell et al., 1999; Mazaleyrat et al., 2003). The temperature-sensitive phenotypes of Su(dx) and Dx suggest roles in temperature compensation of Notch activity. Here, we identify a network of competing endocytic routes mediated by Su(dx) and Dx that positively and negatively regulate N activation; have distinct requirements for ligands, membrane sterol, and endosomal trafficking components; and have different temperature sensitivities. We demonstrate that this trafficking network acts as a robustness module, superimposed on the core N signaling mechanism, to extend the environmental range of normal *Drosophila* development at both upper and lower temperature extremes. Our analyses thus offer interesting insights into mechanisms underlying interactions between genes and environment.

## Results

### Opposing Temperature Dependencies of Notch Signaling by Different Routes

**Figure 1:**
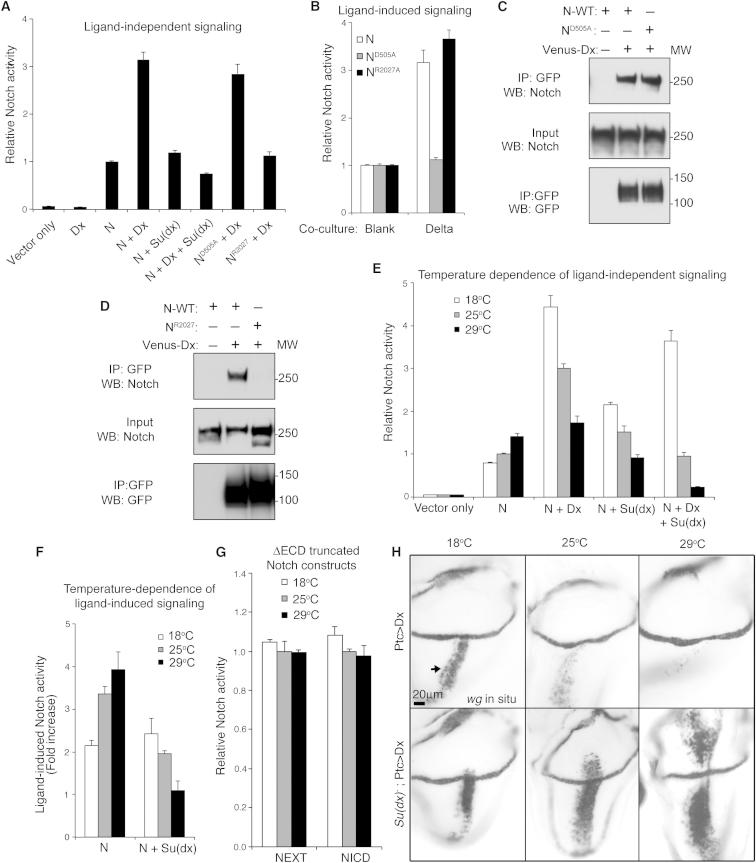
Distinct Mechanisms of Notch Activation Have Opposing Temperature Dependencies (A) Ligand-independent signaling induced by Dx is reduced by Su(dx) coexpression. (B) N^D505A^ prevents Dl-induced signaling. (C and D) Coimmunoprecipitates: Dx binds N^D505A^ (C) but not N^R2027^ (D). (E) Opposite temperature dependence of basal and Dx-induced N signaling. Su(dx) expression increases basal N signal at low temperatures but decreases signal at high temperatures. (F) Dl-induced signaling (fold change) increases with temperature. Su(dx) reduces signal more effectively at high temperatures. (G) Signaling from extracellular truncated N constructs NEXT and NICD is unaffected by temperature. (H) In situ for *wingless* expression in wing imaginal discs marks N signaling (discs shown dorsal up, ventral below). Dx expressed along anterior-posterior compartment boundary using ptc-Gal4. In WT discs, Dx-activated N signaling (arrow) becomes weaker at higher temperatures. In *Su(dx)* mutant discs, the temperature dependency of Dx activity is reversed. Data in (A), (B), (E), (F) and (G) are displayed as means ± SEM, p < 0.05 (minimum of n = 3) for all comparisons stated in legend (Student t test). See also Figure S1.

N signaling can be activated through cell surface interactions with ligand or by ligand-independent trafficking of the full-length receptor to the late endosome and lysosome. We confirmed this separate activation capability in a cell culture N-dependent luciferase reporter assay (Figures 1A and 1B). When N is transfected into S2 cells alone, there is a basal level of signaling that is significantly increased by coexpression with Dx. This increase is prevented by coexpression with Su(dx) (Figure 1A). The ligand binding site mutation N^D505A^ (Whiteman et al., 2013) eliminates the response to Delta (Dl) ligand but binds and is activated by Dx (Figures 1A–1C). In contrast, N^R2027A^ does not bind Dx (Allgood and Barrick, 2011; Figure 1D) and is not activated by Dx expression, but the signaling response to the Dl is unaffected (Figures 1A and 1B). We conclude, therefore, that the binding of Dx to N does not facilitate the ligand-induced component of N activation but instead acts separately to induce signaling through a parallel activation mechanism.

**Figure S1:**
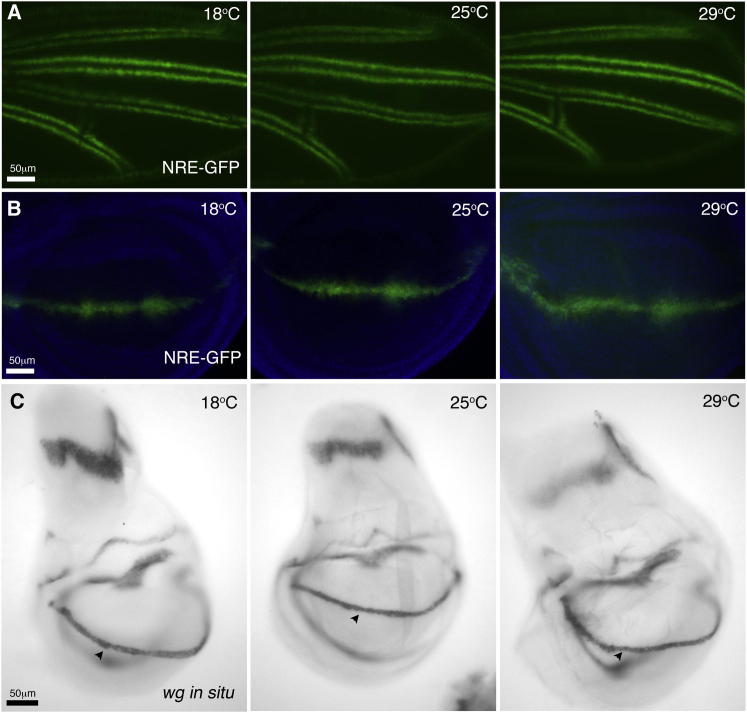
Notch Signaling Is Robust to Temperature Variation, Related to Figure 1 (A) N signaling marked by a Notch response element (NRE)-GFP reporter (green) at 18, 25 and 29^o^C along presumptive vein borders in staged pupal wings (equivalent to 32 hours AP at 25^o^C). (B) Late third instar imaginal discs (DAPI stained, blue) showing NRE-GFP expression along Dorsal-Ventral boundary at 18, 25 and 29^o^C. (C) Late third instar imaginal discs from larvae raised at 18, 25 and 29^o^C and in situ stained for *wingless*, a N responsive gene at the Dorsal-Ventral boundary (arrow head).

In *Drosophila,* N signaling is relatively stable over a wide physiological temperature range (Mazaleyrat et al., 2003; Figure S1 available online). However, the loss of Dx results in insufficient N signal for normal development, and this deficit becomes more severe as temperatures increase (Wilkin et al., 2008). A possible explanation for this temperature sensitivity could be that it reflects changing requirements for Dx-regulated signaling to supplement ligand-induced signaling at different temperatures. If the Dx-dependent component of the N signal normally increased with temperature in WT flies, then this might explain the temperature sensitivity of N signaling when *dx* is removed. However, instead we found that Dx-induced signaling was more effective at low temperatures, while ligand-induced signaling increased with temperature (Figures 1E and 1F). We found that the basal level of N signaling, when N was transfected into S2 cells alone without Dx and without ligand exposure, also increased with temperature. In contrast, N signaling induced by expression of extracellular-truncated and activated forms of N (NEXT, NICD) was not affected by temperature (Figure 1G). This shows that it is the signal initiation mechanisms rather than downstream events that are temperature sensitive. We reasoned, therefore, that to explain temperature sensitivity of *dx* mutant phenotypes, there must be additional temperature-dependent downregulatory mechanisms that are derepressed in the absence of Dx. Because of the strong genetic interactions between *Su(dx)* and *dx*, we tested whether N downregulation by Su(dx) was dependent on temperature. When Su(dx) was expressed in S2 cells at 18°C, it unexpectedly increased basal N signaling (Figure 1E). At higher temperatures, Su(dx) became increasingly effective at reducing the basal N signal and also the signaling induced by ligand or by Dx (Figures 1E and 1F). When we expressed Dx in wing imaginal discs, we found an inverse temperature dependence similar to that observed in cell culture (Figure 1H). When we expressed Dx in *Su(dx)* mutant wing discs, the temperature dependence of the signaling was reversed (Figure 1H). Su(dx) can, therefore, act to prevent excessive N signaling at high temperature. Thus, we have identified different temperature-dependent positive and negative regulatory components of N signaling that, when combined together, could provide a temperature compensation mechanism ensuring that stable signaling is maintained in varying conditions.

### Su(dx) and Deltex Regulate Notch Endocytosis through Distinct Trafficking Routes with Different Temperature Dependencies

**Figure 2:**
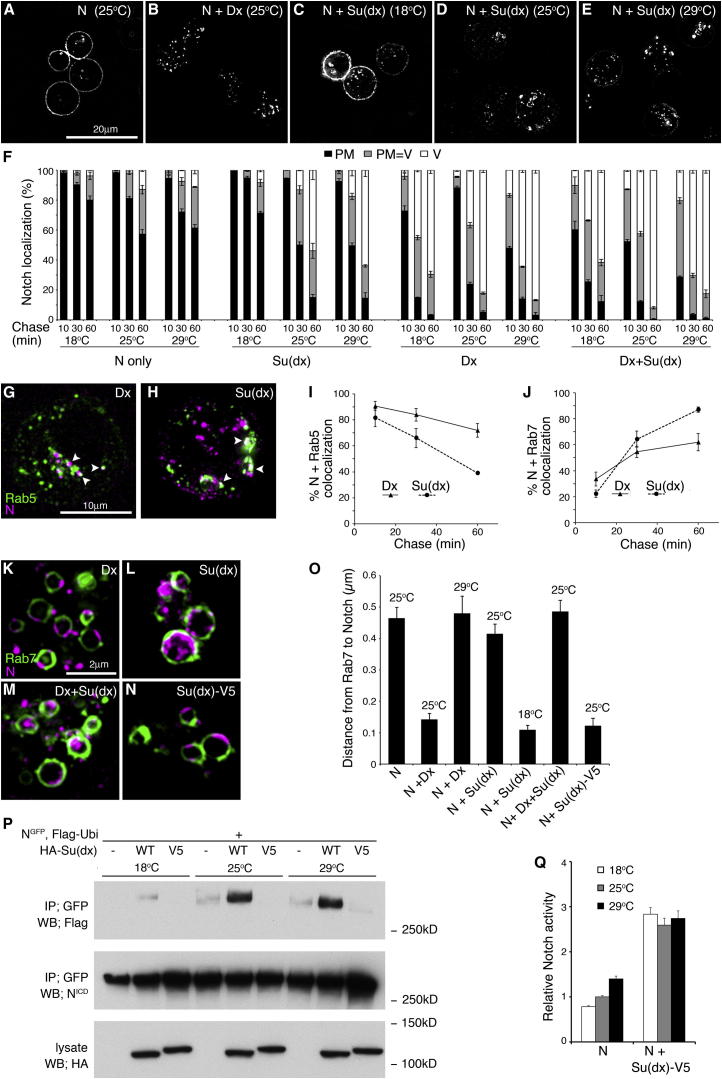
Dx and Su(dx) Induce Notch Endosomal Trafficking with Distinct Temperature Sensitivities (A and B) Dx coexpression promotes N endocytosis. (C–E) N endocytosis resulting from coexpression with Su(dx) increases with temperature. (F) Quantification of temperature dependence of N endocytosis. Localization was scored after indicated chase times as mostly plasma membrane (PM), plasma membrane and vesicular (PM = V), and mostly vesicular (V). Basal N endocytosis and Su(dx)-induced endocytosis increase with temperature. Dx-induced N endocytosis is markedly less sensitive to temperature, as is N endocytosis when Dx and Su(dx) are coexpressed. (G and H) Endocytosed N (purple) colocalization with Rab5-GFP (green) indicated by arrowheads after coexpression with Dx (G) and Su(dx) (H). (I and J) Time course of N progression through Su(dx) and Dx-induced N endocytic pathways. (K) N localizes to the edge of Rab7-GFP-marked vesicle when coexpressed with Dx. (L and M) N in Su(dx)-expressing cells (L) or Su(dx) + Dx-expressing cells (M) is localized within Rab-7-GFP-marked vesicles. (N) Su(dx)V5 induces N endocytosis, but N is localized to Rab7-GFP-marked vesicle limiting membrane. (O) Distance between Rab7-GFP and peak N localization in late endosomes. Increased value represents increased internalization within Rab7-GFP-marked vesicle. Su(dx) expression or increased temperature overcomes ability of Dx to retain N to the edge of the Rab7-marked limiting membrane. Su(dx)V5 prevents N transfer from the late endosomal limiting membrane. At 18°C, Su(dx) is less effective than at 25°C at transferring N into late endosome lumen. (P) Su(dx) promotes temperature-dependent ubiquitination of Notch, but Su(dx)V5 has no ubiquitination activity. (Q) Su(dx)V5 increases N signaling independently of temperature. Data in (F), (I), (J), (O), and (Q) are displayed as means ± SEM (n = 3, minimum 60 cells or vesicles scored per repeat), p < 0.05 for all differences stated in legend (Student t test). See also Figure S2.

**Figure S2:**
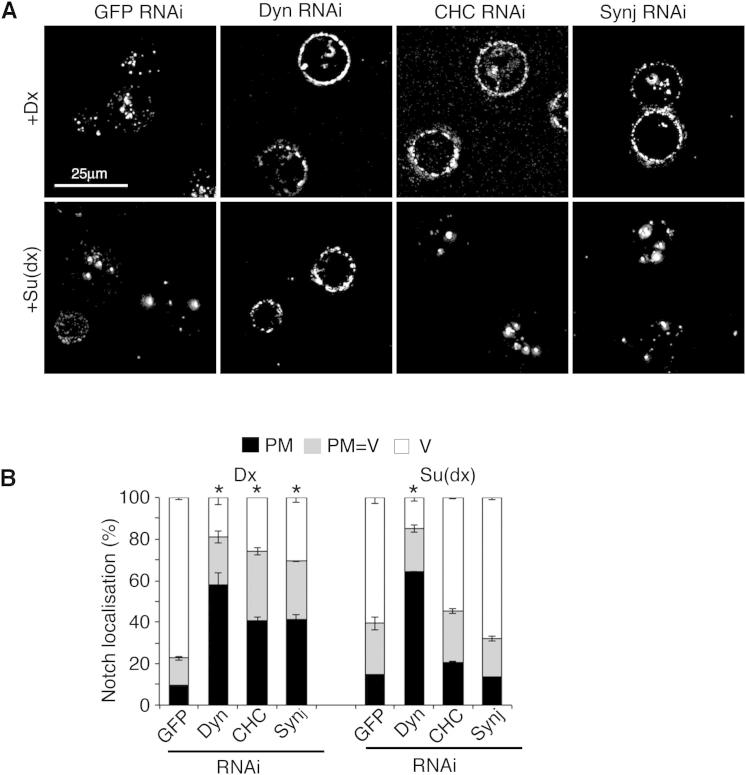
Dx and Su(dx) Induce Notch Endocytosis by Different Routes, Related to Figure 2 (A) Using N antibody uptake assay, Dx induced internalisation of Notch is reduced compared to control by Dynamin, Clathrin Heavy Chain and Synaptojanin RNAi (upper panels in B). In contrast Su(dx) induced Notch endocytosis is prevented by Dynamin but not Clathrin Heavy Chain or Synaptojanin RNAi (lower panels). (B) Scoring of Notch localisation as % cells with mainly plasma membrane localisation (PM), vesicular localisation (V) or a mixture of plasma membrane and vesicular (PM=V). Data displayed as mean ±SEM from minimum of 3 repeats, each scoring minimum of 60 cells, ^∗^ indicates p<0.05 compared to GFP RNAi control (Student t-test).

We next investigated the underlying causes of the temperature-sensitive outcomes of Su(dx) and Dx on Notch signaling. When cells were transfected with N alone, there was a low background level of N endocytosis, which increased with temperature (Figures 2A and 2F). Su(dx)-induced N endocytosis also increased with temperature (Figures 2C–2F). In contrast, Dx-induced endocytosis was independent of temperature (Figures 2B and 2F). Su(dx) or Dx-induced endocytosis both resulted in N trafficking through Rab5- and Rab7-positive early and late endosomal compartments (Figures 2G–2L). As previously observed in vivo (Wilkin et al., 2008), Dx caused N to be retained on the edge of Rab7-green fluorescent protein (GFP)-marked vesicles when expressed at 25°C (Figures 2K and 2O). However, when the temperature was increased to 29°C, endocytosed N was diverted from the limiting membrane into the internal lumen of the vesicles (Figure 2O). In contrast, Su(dx) expression at 25°C led to endocytosed N being localized internally to Rab7-GFP staining (Figures 2L and 2O). We investigated if Su(dx) and Dx compete to regulate N localization in the late endosome. When the two proteins were coexpressed at 25°C, N was localized to the internal lumen. Thus, Su(dx) activity overrides the effect of Dx on N localization in the late endosome (Figures 2L, 2M, and 2O). We next determined whether transfer of N to the internal lumen by Su(dx) was also temperature dependent. When Su(dx) was expressed, the proportion of N located to the internal lumen of the endosomes was decreased as the temperature was lowered to 18°C (Figure 2O). Su(dx) also promoted the ubiquitination of N in a temperature-dependent manner (Figure 2P). We next tested the effect of expressing Su(dx)V5, a construct carrying a C-terminal epitope tag that impairs HECT domain activity (Salvat et al., 2004). Su(dx)V5 was equally effective as Su(dx) at promoting N endocytosis (data not shown), but N was retained on the endosome’s outer edge (Figures 2N and 2O). We found that Su(dx)V5 did not promote N ubiquitination, even at higher temperatures (Figure 2P). Consistent with these results, Su(dx)V5 was able to stimulate N activation without showing any temperature dependence (Figure 2Q). These data show that N entry into the endosomal lumen is a distinct temperature-sensitive HECT domain-dependent step, and they also explain how Su(dx) can act positively on N at low temperature and more negatively as temperatures increase. The different temperature dependencies of N endocytosis induced by Dx and Su(dx) suggested that N may be trafficked by distinct routes. We found that both Dx and Su(dx) induced Dynamin (Dyn)-dependent endocytosis of N. However, only Dx-induced endocytosis was suppressed by RNA interference (RNAi) knockdown of Clathrin or Synaptojanin (Figure S2), the latter also being associated with Clathrin-mediated endocytosis (Schmid and Mettlen, 2013).

**Figure 3:**
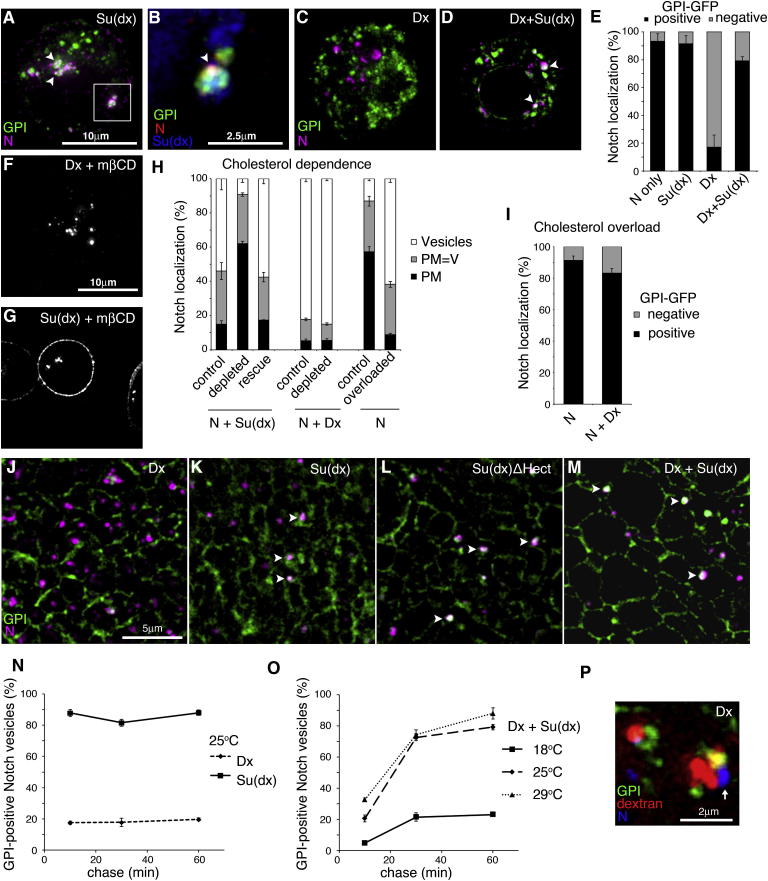
Su(dx) and Dx Regulate Notch Trafficking by Distinct Endocytic Routes (A) When coexpressed with Su(dx), endocytic N (purple) extensively colocalizes with GPI-GFP (green, arrowheads). (B) Enlarged region boxed in (A) showing additional colocalization of Su(dx) (blue, arrowhead). (C) When coexpressed with Dx, endocytosed N does not colocalize with GPI-GFP. (D) Combined expression of Su(dx) and Dx partially relocalizes N into GPI-GFP-marked vesicles (arrowheads). (E) N-positive vesicles were scored as percent GPI-GFP positive or negative. (F) Dx-induced N endocytosis is unaffected in S2 cells treated with mβCD to deplete cholesterol. (G) Su(dx)-induced N endocytosis is suppressed in cholesterol-depleted cells. (H) N localization scored as mostly plasma membrane (PM), plasma membrane and vesicular (PM = V), and mostly vesicular (V) at different cholesterol levels. Cholesterol depletion suppresses Su(dx)-induced N endocytosis, and this is rescued by reloading cells with cholesterol. Dx-induced N endocytosis is unaffected by cholesterol depletion. Overloading of cells with cholesterol induces N endocytosis. (I) Cholesterol overload promotes N endocytosis into GPI-positive vesicles even when Dx is expressed. (J–M) In (J), N (purple) does not colocalize in wing imaginal disc epithelial cells with GPI-GFP (green) when Dx is expressed but does colocalize with GPI-GFP (arrowheads) following expression of Su(dx) (K) or Su(dx)-ΔHECT (L) or after coexpression of Su(dx) with Dx (M). (N and O) Time course of endocytosed N and GPI-GFP localization. (N) In S2 cells, Dx-induced N endocytosis is predominantly through GPI-GFP negative vesicles, while Su(dx) drives N endocytosis through GFP-GFP-positive compartments. (O) Temperature increases the localization of Notch to GPI-GFP-positive compartments when Su(dx) and Dx are coexpressed. An increased proportion of N and GPI-GFP colocalization is observed after longer chase periods. (P) N (blue), endocytosed after Dx expression is localized (arrow) in an endocytic vesicle, marked with Dextran (red) immediately adjacent to a GFP-GPI-marked compartment. Data in (E), (H), (I), (N), and (O) are displayed as means ± SEM (n = 3, minimum of 60 vesicles or cells scored per repeat), p < 0.05 for all differences stated in legend. See also Figure S3.

**Figure S3:**
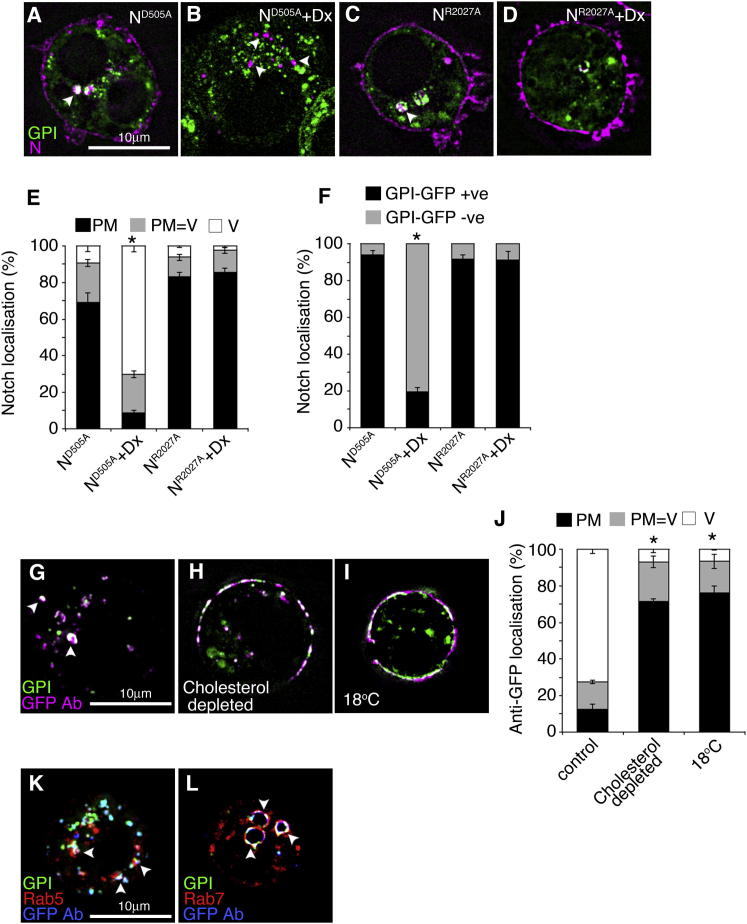
Endocytosis through GPI-Protein Positive and Negative Routes in S2 Cells, Related to Figure 3 (A–D) Immunostained cells after Notch antibody uptake endocytosis assay (Notch, purple; GPI-GFP green). (A) Basal Notch endocytosis of N^D505A^ is into GPI-GFP positive vesicles. (B) Dx expression induces endocytosis of N^D505A^ through GPI-GFP negative vesicles. (C and D) Basal Notch endocytosis of N^R2027A^ is into GPI-GFP positive vesicles (C) and this is not altered by Dx expression (D). (E) Quantification of N localisation as % cells with mainly plasma membrane localisation (PM), vesicular localisation (V) or a mixture of plasma membrane and vesicular (PM=V). (F) % Notch containing vesicles which are GPI-GFP positive or negative. Data in E,F displayed as mean ± SEM from minimum of 3 repeats, each scoring minimum of 60 cells or vesicles per repeat, ^∗^ indicates p<0.05 compared to respective controls (Student t-test). (G–I) GFP antibody uptake assay of GPI-GFP endocytosis (GFP-total green; GFP antibody, purple). (G) In control cells at 25^o^C, most pulse labelled GFP is endocytosed (arrowheads). (H and I) GPI-GFP endocytosis is reduced by cholesterol depletion (H) or by lowering the temperature to 18^o^C (I). (J) Quantification of anti-GFP localisation in endocytic uptake assay shown as % cells with mainly plasma membrane GFP localisation (PM), vesicular localisation (V) or a mixture of plasma membrane and vesicular (PM=V). Data displayed as mean ± SEM from minimum of 3 repeats, each scoring minimum of 60 cells per repeat, ^∗^ indicates p<0.05 compared to respective controls (Student t-test). (K and L) Endocytosed GPI-GFP traffics through Rab5 (K) and Rab7 (L) positive vesicles. (Total-GPI-GFP, green; GFP antibody marking endocytosed GFP (blue); Rab5-RFP (K) or Rab7-RFP (L), red). GFP colocalisation with Rab5 or Rab7 indicated with arrowheads.

Work in mammals has previously identified differences of temperature sensitivity between lipid raft-dependent and raft-independent endocytic routes involved in antigen processing at core versus cooler peripheral locations in the body (Katkere et al., 2010). Glycophosphatidylinositol (GPI)-anchored proteins are markers for such distinct membrane microdomains (Levental et al., 2010). At 25°C, when Su(dx) was expressed, the endocytosed N was predominantly localized, with Su(dx), within GPI-GFP-positive endocytic compartments (Figures 3A, 3B, and 3E). In contrast, Dx expression caused N to localize in GPI-GFP-negative compartments (Figures 3C and 3E). Coexpression of Su(dx) with Dx diverted N to the GPI-GFP-positive compartment (Figures 3D and 3E). To show that a ligand interaction was not required for Notch endocytic entry, we examined the endocytosis of the different N mutant constructs. When expressed alone, WT N and both of the mutant N constructs were similarly endocytosed at a basal rate into GPI-positive compartments (Figures 2A and 3E; Figure S3). Dx was able to drive endocytosis of ligand-binding defective N^D505A^ into the GPI-protein-negative endocytic pathway but, as expected, had no effect on N^R2027A^ (Figure S3).

GPI-enriched membrane domains are associated with sterol molecules (Levental et al., 2010). Insects lack the cholesterol biosynthetic pathway and acquire sterols from their diet. In cell culture, cholesterol is provided in the growth medium. Depleting cells of cholesterol using methyl-β-cyclodextrin (mβCD) had no effect on Dx-induced endocytosis but reduced the endocytosis induced by Su(dx) (Figures 3F–3H). Replenishing of media with cholesterol rescued this effect (Figure 3H). Cholesterol depletion also strongly reduced entry of GPI-GFP into the cell (Figure S3). We also tested the effects of overloading cells with cholesterol and found increased basal levels of N endocytosis (Figure 3H). Increased cholesterol further competed with Dx-induced endocytosis to divert N into the GPI-associated vesicular trafficking pathway (Figure 3I). Thus, trafficking through the cholesterol-dependent route can compete with Dx-induced N endocytosis. Similar differences in Dx- and Su(dx)-induced trafficking of N were observed in wing imaginal discs (Figures 3J–3M).

We next examined the competition between Su(dx) and Dx coexpression at different temperatures during a time course of N endocytic uptake in S2 cells. When we expressed Su(dx) or Dx separately at 25°C, endocytosed N was localized respectively in predominantly GPI-GFP-positive or -negative vesicles throughout the time course (Figure 3N). When we coexpressed Su(dx) and Dx at 25°C, the endocytosed N was initially mainly localized to GPI-negative compartments and then, at later time points, became redistributed to GPI-positive vesicles (Figure 3O). This suggests that N can be sorted into GPI-associated vesicles after its initial endocytosis. Indeed, we found that Dx-endocytosed N was often localized adjacently to GPI-GFP-positive domains on the edge of larger dextran-labeled vesicles (Figure 3P) and that endocytosed GPI-GFP was localized to Rab5- and Rab7-positive vesicles (Figure S3). When Su(dx) and Dx were coexpressed at 18°C, less N entered the GPI-positive route initially, and less transferred to GPI-GFP-positive vesicles after longer chase times. In contrast, at 29°C, more N entered the GPI-positive route at the initial time point (Figure 3O). GPI-GFP endocytosis was also temperature sensitive (Figure S3). Thus, changing the temperature altered the balance of competition at different sorting nodes within the regulatory network. Therefore, Su(dx) and Dx compete in a temperature-dependent manner to determine the route and destination of endocytosed N in the cell. The outcome of their opposing activities further regulates whether N is sequestered into the multivesicular body or is retained at the endosome limiting membrane and, hence, its availability for activation.

### Distinct Endosomal Dependencies of Notch Signal Activation Initiated through Different Mechanisms

**Figure 4:**
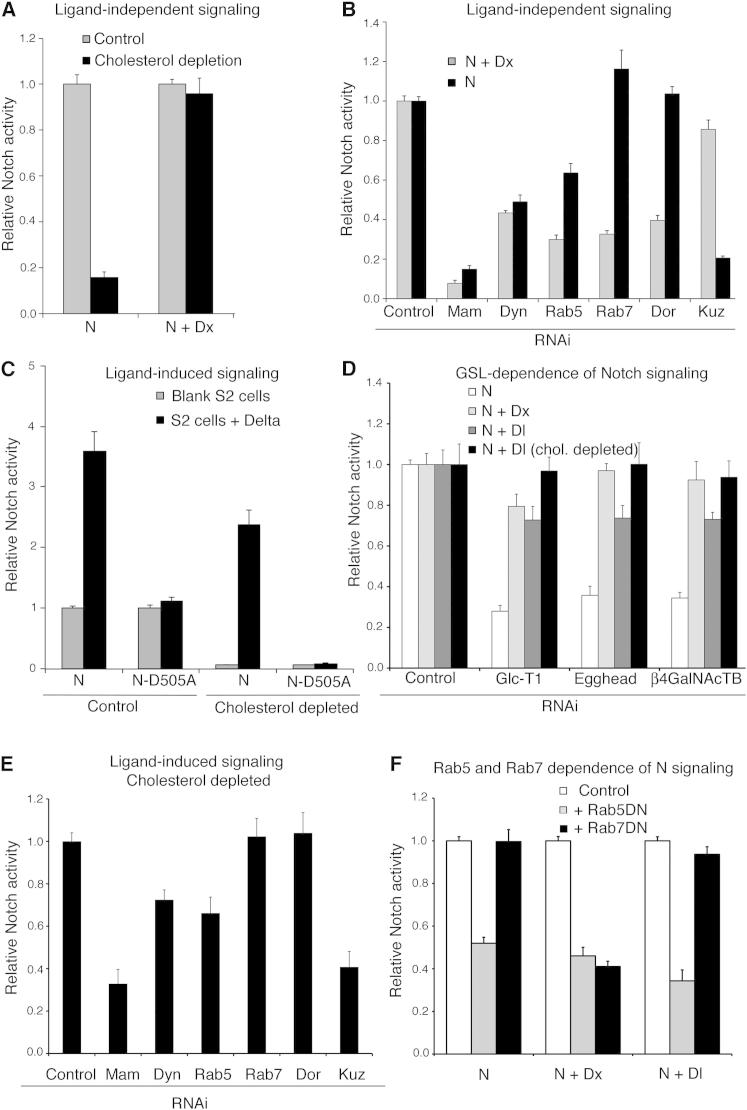
Distinct Requirements for Notch Signaling Initiated by Different Mechanisms (A) Basal N signaling in S2 cells is reduced by cholesterol depletion, but Dx-induced N signaling is unaffected. (B) Basal and Dx-induced N signals are reduced by RNAi of Mam, Dyn, and Rab5, but only Dx-induced signaling is reduced by RNAi of Rab7 and Dor. Basal, but not Dx, signal depends on Kuz. (C) N signaling in cells exposed to Dl is reduced by cholesterol depletion, but the fold change after ligand-induction is increased. Signaling by the N^D505A^ construct is removed by cholesterol depletion. (D) RNAi knockdown of components of the GSL synthesis pathway preferentially reduces the basal N signal compared to Dx and ligand-induced signal. chol., cholesterol. (E) In cholesterol-depleted cells, ligand-induced N signaling is reduced by RNAi of Kuz, Dyn, and Rab5 but insensitive to Rab7 or Dor RNAi. (F) Rab5DN reduces basal, ligand, and Dx-induced signaling, but only Dx signaling is reduced by Rab7DN. Data are displayed as means ± SEM (minimum n = 3), p < 0.05 for differences stated in the legend (Student t test). See also Figure S4.

**Figure S4:**
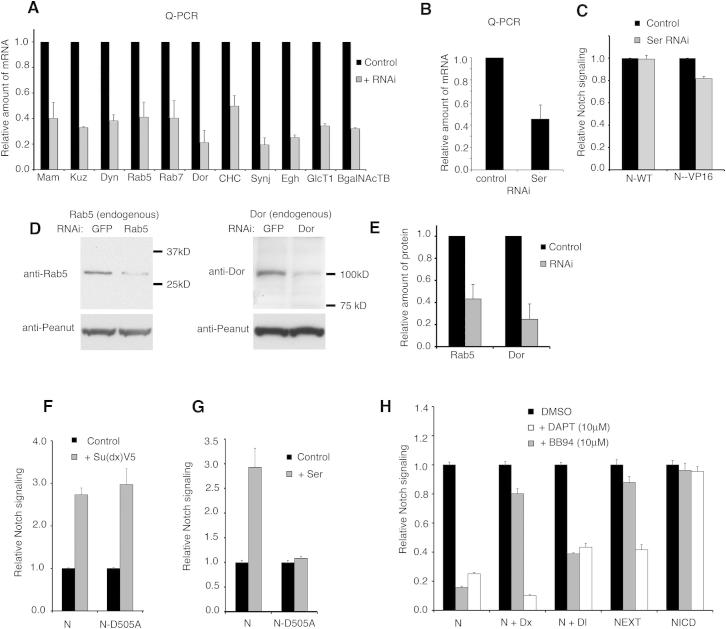
RNAi Knockdown Efficiency and Effect of ADAM 10 Inhibitor on Notch Signaling in S2 Cells, Related to Figure 4 (A) Expression of mRNA of target genes in S2 cells was detected by real time Q-PCR and effect of RNAi knockdown is shown relative to respective normalised control exposed only to GFP RNAi. Expression of Notch is from pMT-Notch transfected cells, the expression of other genes reflects endogenous expression. (B) RNAi Knockdown of Ser expression in S2 cells detected by Q-PCR. (C) Notch signaling luciferase assay. Basal signal through WT Notch is insensitive to Ser RNAi, but signal from N-VP16 shows small but reproducible reduction compared to control (GFP RNAi). (D) Western blots showing relative reduction in protein levels, Rab5 and Dor. Anti-Peanut was used as a loading control. (E) Quantification of western blots. (F–H) Notch signaling luciferase assay. (F) Su(dx)V5 induces signaling similarly through N and N^D505A^ but (G) Ser does not induce signaling through N^D505A^. (H) Kuz inhibitor BB94 blocks basal and Dl-induced signaling but not Dx activation of N. As a control the Presenilin inhibitor DAPT blocks, basal, Dx and Dl-induced signal. Extracellular truncated N controls behave as expected. NEXT is inhibited by DAPT but not BB94, NICD is not affected by either inhibitor. Data in A-C, E-H displayed as means ± SEM for 3 repeats.

Because we had found an opposite temperature dependence of basal compared with Dx-induced N activation in S2 cells (Figure 1E), we suspected different mechanisms of activation were involved. We found that Dx-induced N signaling was unaffected by cholesterol depletion, while basal N activity was suppressed (Figure 4A), suggesting that most of the basal signal arose only by the GPI-protein-positive route. We next used a panel of RNAi knockdowns to probe the underlying mechanisms of N activation arising in different conditions (Figures 4B–4E; Figure S4). Dx-induced signaling was reduced by knockdown of the early endosomal trafficking proteins Dyn and Rab5, and by the late endosomal trafficking components Rab7 and Dor, but not by RNAi against Kuz (Figure 4B). The basal N signal required Dyn, Rab5, and Kuz, but not Rab7 or Dor (Figure 4B). Thus, N signaling can be activated within both cholesterol-dependent and -independent trafficking routes with distinct requirements for endosomal components and Kuz. We investigated if either of the endocytic routes to N activation contributed to ligand-stimulated N signaling. Although total N signaling in the presence of ligand was reduced by cholesterol depletion, the fold change on exposure to ligand was greatly increased (Figure 4C). This suggested that cholesterol depletion had preferentially removed only the background basal signal without affecting the ligand-induced signal. This conclusion was supported by cholesterol depletion eliminating the basal signaling through the ligand binding site mutation N^D505A^. The latter does not respond to either Dl or Serrate (Ser) ligand but does respond to Su(dx)V5, which increases N endocytosis and activation through the GPI-protein-positive route (Figure 1B; Figure S4). Cholesterol-rich membrane microdomains are enriched in glycosphingolipids (GSLs; Levental et al., 2010). We found that knockdown of components of the GSL synthesis pathway had little effect on ligand- or Dx-stimulated N signaling but greatly reduced the basal signal (Figure 4D). Thus, we conclude that the observed basal N signal acts substantially through an endocytic route that requires cholesterol-dependent endocytosis and is thus distinguishable from ligand-induced signaling. S2 cells do not express endogenous N or Dl (Fehon et al., 1990) but have been reported to express Ser (Saj et al., 2010). We found that basal signaling through N was insensitive to Ser RNAi (Figure S4), although we do not rule out some contribution of endogenous Ser to the basal signaling level, as previously reported using a N-VP16 fusion protein (Saj et al., 2010; Figure S4).

**Figure 5:**
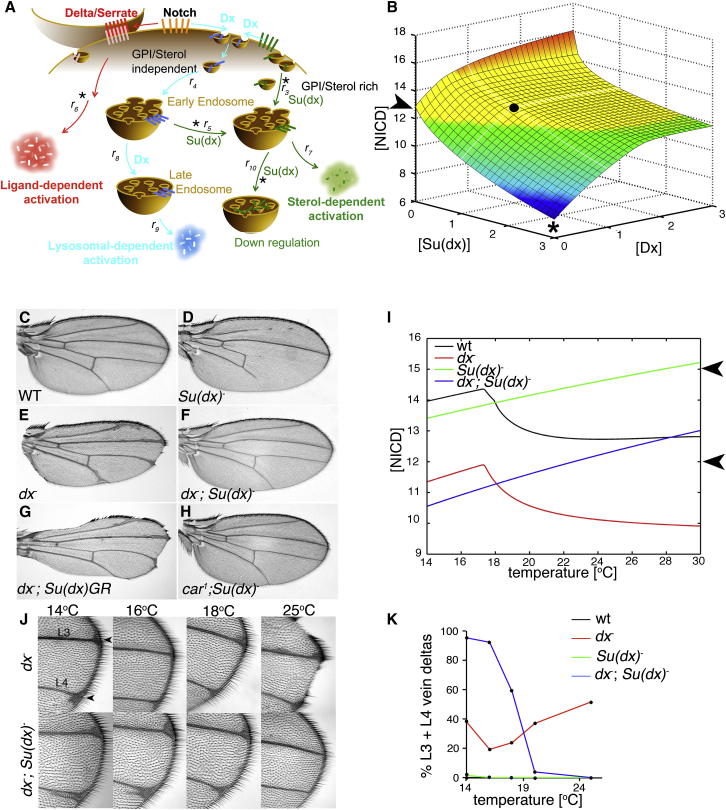
Modeling Consequences of *Su(dx)* and *dx* Mutations on Notch Signaling in the *Drosophila* Wing (A) N endocytic trafficking routes. Asterisks mark experimentally observed temperature-dependent steps. Key fluxes are designated r. (B) Model of the effects of mutations of *Su(dx)* and *dx* on N signaling ([NICD] arbitrary units) in the *Drosophila* wing at 29°C. Arrowhead marks mutual suppression observed in double mutants. Black dot represents WT concentrations of both Dx and Su(dx). Asterisk marks stronger loss of Notch signaling expected from increased Su(dx) in the absence of Dx. Yellow shading indicates expected WT conditions, orange to red shading indicates increased expectation of a Notch gain of function phenotype (wing vein gaps) and green to blue shading indicates increased likelihood of a Notch loss of function phenotype (vein thickening). (C–F) Mutual suppression resulting from combined *Su(dx)* and *dx* mutations restores temperature-sensitive phenotypes of each back to WT at 29°C. (G) Increasing WT *Su(dx)* copy number enhances the *dx* mutant wing phenotype. (H) *Su(dx)* mutant wing phenotype is suppressed by *car*. (I) Simulation of N signaling (NICD) versus temperature in WT and mutant backgrounds. Arrowheads mark expected upper and lower signaling thresholds corresponding to yellow shaded area in (B). (J) *dx* mutant phenotype weakens as temperatures decrease from 25°C down to 16°C but worsen again at 14°C. At 25°C, *Su(dx)* mutants suppress *dx* phenotype, but as temperature is lowered, the loss of *Su(dx)* has less effect on the strength of the *dx* phenotype. At 18°C and below, *Su(dx)* mutation enhances *dx* phenotype. Arrowheads indicate distal thickening on L3 and L4 veins. (K) Percent wings with L3 and L4 vein thickening. The *dx* phenotype was increased at 14°C compared to 16°C (p < 0.05) and reduced at 18°C compared to 25°C (p < 0.001). Enhancement or suppression of *dx* phenotype in *dx;Su(dx)* double mutant flies was observed at different temperatures (p < 0.0001, Fisher’s exact test, n > 40 for each genotype tested). See also Data File S1.

We next investigated the endocytic requirement for ligand-stimulated signaling after the cholesterol-dependent background was removed. The ligand-activated signal was sensitive to reduction of Kuz and to the RNAi of Dyn and Rab5 but not the late endosomal components Rab7 and Dor (Figure 4E). Furthermore, ligand-induced Notch signaling was inhibited by dominant negative Rab5 (Rab5DN) and ADAM10 inhibitor treatment but not by dominant negative Rab7 (Rab7DN) (Figure 4F; Figure S4). The expression of Rab5DN and Rab7DN had similar effects to Rab5 and Rab7 RNAi on basal and Dx-induced signaling (Figure 4F). These differing requirements, therefore, define three distinct routes to Notch activation. Ligand-independent signaling can be sensitive or insensitive to cholesterol depletion, but if the latter then N activation depends on late endosomal trafficking and is independent of Kuz. Ligand-dependent signaling is insensitive to depletion of cholesterol but is not dependent on late endosomal trafficking and requires Kuz. Therefore, these results demonstrate how overall N signaling levels can result from the summation of different activation mechanisms (Figure 5A). The differential temperature sensitivity of different components thus offers a possible mechanism to compensate for environmental fluctuation by altering the proportion of total N signaling that is contributed by different routes to maintain levels within appropriate thresholds.

### Mathematical Modeling of Temperature Compensation of Notch Signaling

To investigate the potential for the identified network of N trafficking routes to confer environmental robustness, we developed a computational model, described by a set of differential equations. These were solved for steady state to investigate the balance of fluxes through the system (Figure 5B; Data File S1). This model comprises a simplified ligand-induced canonical pathway and incorporates the sterol-dependent and independent endocytic trafficking routes that are increased, respectively, by Su(dx) and Dx. It also incorporates the sorting of N by Su(dx) into the multivesicular body away from the late endosomal limiting membrane. Parameters were optimized to simulate the gain and loss of N signaling observed in the fly wing in the absence of *Su(dx)* and *dx* at 29°C and also to simulate the mutual phenotypic suppression resulting when these mutations are combined (Figures 5C–5F; Data File S1). We assumed that changes in N signaling levels are reflected by changes to the penetrance and strength of the observed phenotypes. In our model, reduced N signaling in the *dx* mutant phenotype results in part from reduction of the lysosomal activation mechanism but also from increased Su(dx) downregulation of N activity (Data File S1). The latter arises due to lack of competition with Dx for N trafficking. This model predicts, therefore, that in the absence of Dx, increasing Su(dx) copy number would more strongly downregulate N signaling (Figure 5B). We tested this in vivo and found the prediction to be correct (Figure 5G), while an extra copy of Su(dx) in a WT background had no visible phenotypic effect (data not shown). In turn, the *Su(dx)* mutant phenotype depends, in part, on a lysosomal N activation component (Data File S1). We confirmed this by combining *Su(dx)* with the *car* mutant, which reduces HOPS complex activity, and this suppressed the wing phenotype (Figure 5H). Thus, there is competition between Dx and Su(dx) activities on N.

We then investigated whether the model could simulate observed temperature sensitivities of *Su(dx)* and *dx* mutant phenotypes. The temperature sensitivities of the relevant parameters were optimized to reflect experimentally observed changes in flux (Data File S1). The model simulated the stability of N signaling in WT over a wide temperature range. This temperature insensitivity was brought about by changing the relative contributions of flux through the three different routes to NICD generation (Data File S1). The model was then able to recapitulate the observed temperature-sensitive wing phenotypes of *Su(dx)* and *dx* mutations, which lead to, respectively, stronger gain and loss of N function at increased temperatures (Figure 5B). The model also resulted in a number of counterintuitive predictions. First, it predicted that the temperature sensitivity of the *dx* mutant phenotype would become inverted and that the phenotypes would start to strengthen again as temperatures were reduced below a critical temperature (Figure 5I). This prediction was verified in vivo. We found that, between 16°C and 18°C, *dx* flies had a minimal phenotype, which became stronger and more penetrant at 14°C (Figures 5J and 5K). A second prediction was that, at low temperatures, removing both *Su(dx)* and *dx* would result in stronger N loss-of-function phenotypes than removing *dx* alone (Figure 5I). This prediction was also verified in vivo. We found that, below 20°C, the effect of the *Su(dx)* mutation on *dx* was reversed to enhance rather than suppress the *dx* wing phenotype (Figures 5J and 5K). Thus, at low temperatures, there is a switch to cooperation between Su(dx) and Dx instead of antagonism, and they then act together to support N activity. Our model was thus able to predict and account for different genetic interactions between *Su(dx)* and *dx* mutant phenotypes observed at upper and lower physiological temperature extremes in the fly wing.

### Altering Network Parameters Enables Su(dx) and Deltex to Cooperate in Downregulating Notch

**Figure 6:**
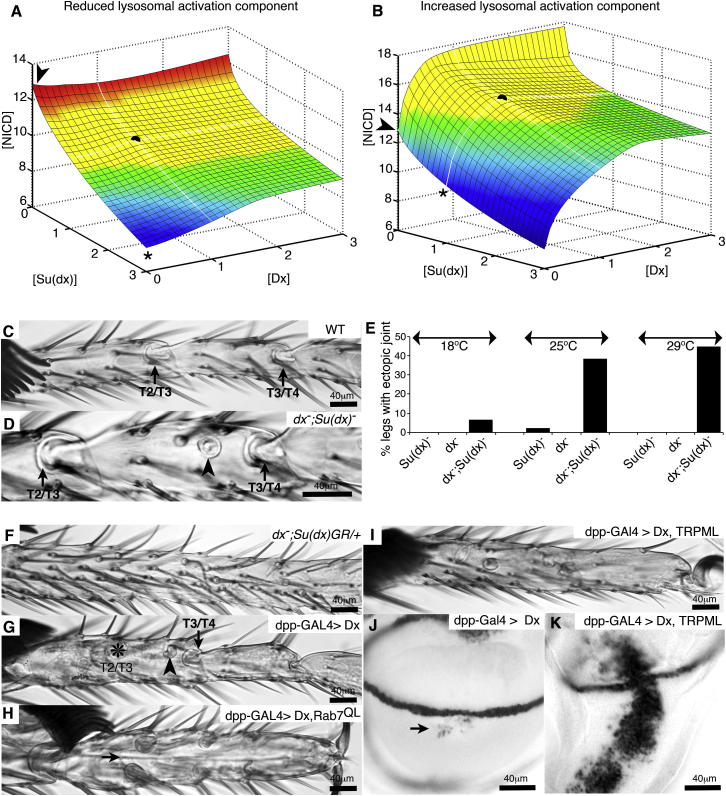
Tuning of Network Parameters Predicts Tissue-Specific Cooperation between Su(dx) and Dx to Downregulate Notch (A and B) Modeling of the combined loss of function of *Su(dx)* and *dx* on N signaling ([NICD] arbitrary units) when lysosomal activation component is reduced (A) or increased (B). These models differ from that shown in Figure 5B only by a 5-fold reduction (A) or 5-fold increase (B) in *k*_*9*_, which determines lysosomal activation component. Black dot represents WT [Dx] and [Su(dx)], color shading is as described in the legend for Figure 5B. (C–E) *Su(dx), dx* mutant combination results in N gain of function. (C) WT leg, tarsal joints between segments T2/T3, and T3/T4 indicated. (D) Extra joint tissue (arrowhead) observed in *dx;Su(dx)*. (E) Percent legs with ectopic joint increases with temperature in double mutants (p < 0.01, Fisher’s exact test, n > 60 legs per genotype). (F) Additional copy of WT *Su(dx)* in *dx* mutant results in loss of joints at 25°C (86.4% legs, n = 66) not seen with additional WT *Su(dx)* copy in WT background (n = 80). (G) Dx expression in WT results in both partial joint loss (asterisk) and ectopic joint material (arrowhead). (H) When active Rab7 (Rab7^QL^) is coexpressed with Dx, joint tissue is lost (arrow). (I) TRPML coexpression with Dx results in loss of joints. (J and K) Coexpression of TRPML with Dx increases *wingless* expression in wing discs compared to Dx alone (arrow). TRPML expression alone has no effect (data not shown). See also Data File S1.

We found that the predicted outcome of removing both Su(dx) and Dx functions together was critically dependent on the relative efficiency in WT of N activation in the lysosome versus its inactivation. We identified three classes of model represented by low, intermediate, and high contributions of the lysosomal activation component (Figure 5B; Figures 6A and 6B; Data File S1) reflected in the modeling by changes only to the parameter *k*_9_ (Figure 5A; Data File S1). The mutual suppression of *dx* and *Su(dx)* wing phenotypes was only simulated at an intermediate contribution of lysosomal N activation, as depicted in Figure 5B. When a WT situation was modeled with a reduced contribution of lysosomal activation, then the predicted consequence of removing both *Su(dx)* and *dx* was to more strongly increase N signaling compared to removing *Su(dx)* alone (Figure 6A). In this circumstance, the net contribution of Dx-regulated N endocytosis to overall signaling levels would be negative rather than positive. In contrast, in a model where lysosomal N activation made a larger contribution to signaling levels, the simulated loss of *Su(dx)* did not strongly suppress the *dx* mutant phenotype (Figure 6B). Thus, the observed mutual genetic suppression in the wing seems to arise as a special case from a larger range of possible interactions.

We investigated mutant flies that were homozygous for both *dx* and *Su(dx)* to determine if any of the predicted alternative outcomes were present in other tissues apart from the wing. In adult flies lacking both *dx* and *Su(dx)*, we identified ectopic leg joint tissue (Figures 6C–6E), which is an N gain-of-function phenotype (Bishop et al., 1999). This phenotype resulted from the combined removal of both genes because extra joint phenotypes were seen infrequently in *Su(dx)* mutant flies and not present in *dx* mutants alone (Figure 6E). Thus, the observed genetic interaction closely resembled the outcome of the model depicted in Figure 6A. As in the wing, the leg phenotypes were temperature dependent and became less penetrant as temperatures were reduced (Figure 6E), a trend also observed in the computer simulations (Data File S1). We tested a further interesting prediction of this leg-type model, which suggested that increasing the gene copy number of WT *Su(dx)* could switch the effective contribution of Dx to N signaling from negative to positive (Figure 6A). In a WT background, an extra copy of *Su(dx)* gave no phenotype in the legs, but when *dx* was also removed, there was a loss of joint phenotype that was associated with reduced N activity (Figure 6F). Thus, when the *Su(dx)* gene copy number is raised, the net effect of removing *dx* on N signaling is negative, whereas in a background lacking any *Su(dx)*, the consequence of removing *dx* on N is to upregulate signaling. Therefore, although loss of *dx* alone has little effect on leg development, its loss leaves N regulation balanced on a knife edge that can easily lead to breaching of either upper or lower thresholds of signaling.

We next investigated whether the capacity for N activation in the lysosomal route differed in the wing and the leg, as would be expected from the models represented by Figures 5B and 6A. We tested the ability of Dx to activate N in the leg by expression with dpp-Gal4. We found that Dx induced a mixed phenotype with both ectopic leg joints, indicating increased N activity, and loss of joints indicative of reduced N (Figure 6G). This suggested that the outcome of Dx action to drive N endocytosis in the leg was less robust compared to the wing and could tip toward either a positive or negative outcome. We next investigated the consequences of stimulating late endo-lysosomal fusion by coexpression of Dx with Rab7QL, a constitutively activated mutant form of Rab7. In the wing, this combination strongly upregulates N signaling (Wilkin et al., 2008). However, in the leg, the same combination resulted in a clear loss of leg joints, indicating loss of N signaling (Figure 6H). We obtained similar results by coexpressing Dx with the calcium channel protein TRPML (Transient receptor potential cation channel, mucolipin), which also stimulates endo-lysosomal fusion (Venkatachalam et al., 2013). The combination produced loss of joints in the leg but strongly enhanced N signaling in the wing (Figures 6I–6K). Therefore, the endo-lysosomal regulation of N has a more limited capacity to allow N activation in the leg compared to the wing, consistent with the model depicted in Figure 6A.

**Figure 7:**
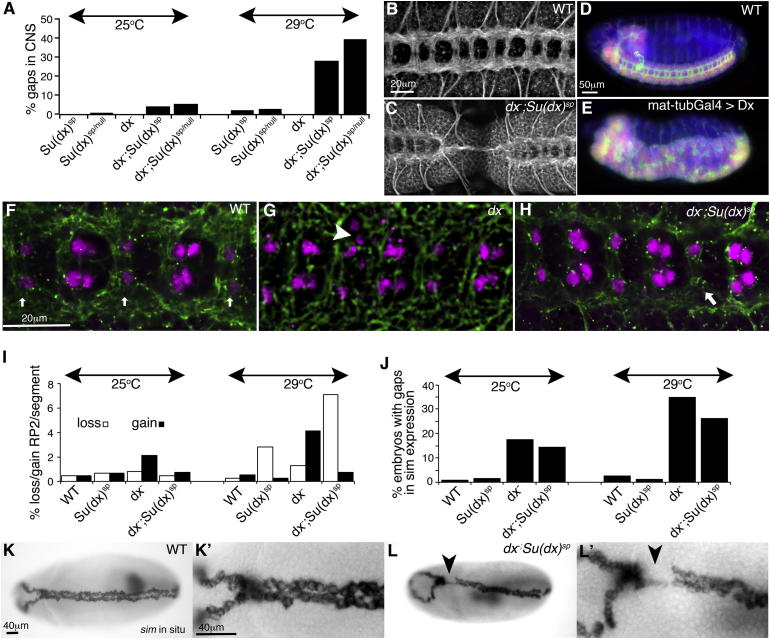
Modularity of Network Masks Critical Roles for Su(dx) and Dx during *Drosophila* Embryogenesis (A) *dx;Su(dx)* maternal/zygotic mutant embryos show more frequent and temperature-dependent gaps in the central nervous system (CNS) compared to *Su(dx)* or *dx* (p < 0.005, n > 30). (B) Anti-Hrp-stained CNS of WT stage 15-16 embryo. (C) CNS loss in *dx;Su(dx)* embryo. (D) WT stage 15 embryo CNS, anti-ELAV (red), anti-HRP (green), DAPI (blue). (E) Neurogenic phenotype after Dx expression using mat-tubGal4. (F) WT embryo, anti-Eve (purple), anti-Hrp (green). Pairs of RP2 neurons are indicated by arrows. (G) Extra RP2 neurons in *dx* (arrowhead). (H) Loss of RP2 in *dx;Su(dx)* embryo (arrow). (I) RP2 loss in *dx;Su(dx)* at 29°C is more frequent than for either mutant on its own or for *dx;Su(dx)* at 25°C (p < 0.01). A gain of RP2s was observed in *dx* compared to WT at 29°C, p < 0.01 (>230 segments per genotype scored at stage 15/16). (J) Reduced *sim* expression in stage 7-8 *dx* embryos, (p < 0.001, n > 60), with increased penetrance at higher temperature. The *dx* phenotype was not strongly reduced by *Su(dx)*. (K–L′) In situ staining of *sim* in WT (K) and *dx;Su(dx)* (L) embryos. (K′) and (L′) show enlarged images of similar areas of (K) and (L) where arrowheads indicate gap in *sim* expression in (L) and (L′). Statistics by Fisher’s exact test.

Our observations that Su(dx) and Dx could have cooperative functions suggested the two proteins might have a wider developmental requirement than previously anticipated. In *Drosophila* development, there is both a maternal and a zygotic contribution to gene function because of transfer of maternal messenger RNA into the oocyte. To investigate early developmental requirements, we examined embryos from double homozygous mutant parents that were defective in both maternal and zygotic contributions of Su(dx) and Dx (Figure 7). In central nervous system development, *Su(dx)* mutants alone displayed a weakly penetrant, and temperature-sensitive, loss of neurons consistent with increased N activity (Figures 7A and 7I). The *dx* mutation alone had a weakly penetrant gain of neurons at 29°C, consistent with mildly reduced N activity (Figures 7G and 7I). The loss-of-neuron phenotype of *Su(dx)* was substantially increased by mutation of *dx* (Figures 7A–7C, 7F–7I) as predicted by the low lysosomal N activation model in Figure 6A. Overexpression of Dx induced a strongly neurogenic (N loss-of-function) phenotype (Figures 7D and 7E), suggesting a low capacity for activation by the lysosomal trafficking route in the nervous system. We additionally investigated N signaling during midline formation, where we have previously shown that late endosomal trafficking components have a more significant contribution to N signaling levels (Wilkin et al., 2008). N signaling in the midline can be monitored through *single minded* (*sim*) expression and is reduced by mutants of *dx* and HOPS complex components in a temperature-dependent manner (Wilkin et al., 2008). We found that this *dx* mutant phenotype was not strongly suppressed by removing *Su(dx*) (Figures 7J–7L). The latter situation is, therefore, consistent with the prediction made by the model depicted in Figure 6B, which has a higher lysosomal activation component.

The tissue-dependent tuning of network parameters can, therefore, result in widely different consequences of removing both *dx* and *Su(dx)* function, and a counterintuitive functional redundancy between *dx* and *Su(dx)* can emerge from the architecture of the network in which these genes participate.

## Discussion

Here, we used a combined experimental and modeling approach to demonstrate a solution to the problem of temperature compensation, which stabilizes N signaling at both high and low temperature extremes. We identified an unexpected variety of mechanisms by which the N receptor can be trafficked and activated within the cell in different cellular locations with different temperature dependencies. Additional to ligand-stimulated activation, two distinct ligand-independent endocytic routes to N activation were identified. The overall signaling levels can thus be viewed as the sum of a number of component parts. In our model, robustness emerges through temperature-dependent changes in flux through competing trafficking routes, which alters the relative contributions of the component parts of the signal in a compensatory fashion.

A number of studies have revealed roles for N endocytosis in both signal activation and its downregulation (reviewed by Fortini and Bilder, 2009; Baron, 2012), but the nature of the trafficking pathways involved and their relationship to the mechanisms of signal activation have been unclear. In this study, we have shown that N traffics through distinct endocytic routes with different outcomes. Dx-induced N endocytosis occurs through GPI-protein-negative endosomes, is insensitive to cholesterol depletion, and leads to N signaling by the lysosomal activation mechanism, independently of the S2 metalloprotease Kuz. It is interesting that Kuz-independent N activation also results from loss of *Lethal giant discs* and may require lysosomal proteases to remove the N extracellular domain (Schneider et al., 2013). Alternatively, N can enter a GPI-protein-positive and cholesterol-sensitive endocytic route. N can also be activated in the latter route by a mechanism that does not require late endosomal trafficking but is sensitive to reduction of early endosomal trafficking components. Both of these routes had endocytic requirements distinct from the ligand-induced signaling mechanism, revealing a remarkable plurality of means by which N can be activated. The finding that N signaling can be activated by such diverse routes has important implications for understanding and, possibly, specifically ameliorating the mechanisms of misactivation of N in diverse tumors. Recent work has demonstrated the involvement of late endosomal HOPS components in the ligand-independent activation of mouse Notch-1 receptor in HeLa cells (Zheng et al., 2013), indicating that alternate routes to activation are indeed present in mammalian cells. It will, therefore, be informative to identify mechanisms of N misactivation in different contexts using the criteria we have established in this study.

Two components of the trafficking network play a key role in ensuring thermal robustness of N signaling by compensating for increased signaling at high temperature and the decreased ligand-induced activation at low temperature. Su(dx) competes with Dx to divert more N into the GPI-protein-positive endosomal vesicles. Su(dx) additionally acts to limit endosomal N activation by promoting the transfer of N into the multivesicular body. The latter step was found to be a biochemically distinct activity of Su(dx), which, unlike its effects on N endocytosis, requires a functional HECT domain to promote N ubiquitination. N endocytosis by the cholesterol-dependent and -independent endocytic routes had different responses to temperature. Cholesterol-dependent endocytosis into the N degradative route increased with temperature, while the cholesterol-independent N trafficking induced by Dx was insensitive to temperature. Thus, as temperature increases, Su(dx) is more effective at competing with Dx to divert N into the GPI-positive endocytic route and then to promote entry into the multivesicular body. The HECT domain activity of Su(dx) also acts as a temperature-dependent switch regulating N ubiquitination. The latter is reduced as temperatures are lowered and N is retained on the endosomal limiting membrane. This means that, at low temperatures, Su(dx) acts to increase rather than decrease the basal activity of N signaling. When superimposed on the core ligand-induced N activation pathway, the flexible trafficking network has the ability to both positively and negatively tune overall signaling levels through changes in rates and directions of endocytic flux. Thus, signaling can be kept within appropriate thresholds across a range of different temperatures. This network solution provides a view of developmental signaling more akin to metabolic pathway integration; for example, different routes to ATP generation.

Mathematical modeling provided a compelling argument that temperature insensitivity of N signaling could emerge in vivo through the operations of the N trafficking network. As well as being capable of simulating the temperature-dependent consequences of removing *Su(dx)* or *dx* function, the model was supported by verification of several remarkable predictions regarding unusual context-dependent genetic interactions between the two genes. Both *Su(dx)* and *deltex* genes have been previously associated with relatively mild N loss and gain-of-function mutant phenotypes that are mutually suppressive when combined in the same fly (Xu and Artavanis-Tsakonas, 1990; Fostier et al., 1998). Therefore, the biological necessity to maintain both genes in the genome has hitherto been obscure. Our findings now explain this paradox. The mutual suppression between these mutations is actually a special case from a range of possible parameter-dependent outcomes. Exploration of these different outcomes revealed how an apparent redundant requirement for *Su(dx)* and *dx* in both embryo and adult tissues can emerge from the modularity of the network in which they participate. Through mathematical modeling and experimental observations, we found that cooperation rather than antagonism between Dx and Su(dx) function could emerge at low temperatures to sustain N signaling. An alternative outcome of the model was predicted if the contribution from the lysosomal activation component to overall N signaling levels in WT tissue was reduced. In this case, the Dx contribution was switched to cooperate with Su(dx) to restrict N signaling. This predicted outcome was confirmed in vivo by mutant analyses of leg developmental phenotypes in which the requirement to restrain N signaling was found to become more significant at higher temperature. Intriguingly, the net contribution of Dx to N signal levels could switch from negative to positive, depending on *Su(dx)* gene copy number. Thus, in the absence of *dx*, N signaling is at a tipping point, easily able to gravitate beyond critical high or low signaling thresholds in response to genetic background or unfavorable environmental conditions. This loss of robustness helps explain the extreme sensitivity of *dx* mutant flies to changes in copy number of N pathway components (Xu and Artavanis-Tsakonas, 1990). The intricate interplay of cooperative and antagonistic interactions that we have revealed to occur between the two N-binding ubiquitin ligases is remarkable and, to our knowledge, unprecedented in the literature. The regulatory effects of Dx and Su(dx) are highly dependent on the status of the remainder of the network, and their overall contribution to development can only be comprehended at this systems level. The outcome is that Su(dx) and Dx can act in cooperation to sustain or limit N signaling at low and high extremes, respectively, of the physiological temperature range, thus extending the temperature range over which normal development can occur. It may be significant that other signaling receptors are positively and negatively regulated by different trafficking routes (Sigismund et al., 2008; Di Guglielmo et al., 2003). Thus, regulatory mechanisms similar to those described in this work might conceivably operate to stabilize other developmental pathways against temperature fluctuation.

In summary, this study provides a valuable insight into mechanisms by which the interplay between genes and environment can be manifested. Since endocytosis is modulated by numerous environmental and physiological inputs, its role in environmental tuning of signaling may extend beyond temperature compensation. For example, changes in nutrient availability, dietary composition, cholesterol levels, hypoxia, and other cellular stresses (as well as aging) may all affect endocytic functions with potential impact on N activity. Our model now provides a theoretical framework by which to explore how different environmental and other regulatory inputs can be integrated with the core signaling mechanism to result in adaptive—or, possibly, maladaptive—outcomes on the development, maintenance, and health of the organism. Our comprehension of the extraordinary variety of routes available for activation of N will further inspire reevaluation of numerous regulatory phenomena and provide insights into means of misregulation of N in disease.

## Experimental Procedures

### *Drosophila* Stocks

*Drosophila* stocks used are listed in the Extended Experimental Procedures.

### S2 Cell Culture and Immunohistochemistry

S2 cells (Invitrogen) were grown in Schneider’s medium (Invitrogen), with 10% fetal bovine serum (FBS) (Hyclone) and 1% penicillin-streptomycin (Sigma), and transfected using Effectene (QIAGEN). Expression constructs utilized the CuSO_4_-inducible pMT vector (Invitrogen) and are listed in the Extended Experimental Procedures. Immunostaining of fixed cells was performed using protocols described in the Extended Experimental Procedures. For N and GPI-GFP uptake assay, S2 cells, grown on coated coverslips, were incubated with anti-Notch ECD or anti-GFP for 15 min on ice, washed with ice-cold S2 medium, and chased for up to 60 min at 25°C with or without 0.5 mM lysine-fixable Texas Red-Dextran-3000 (Molecular Probes). Cells were fixed, permeabilized, and stained as described earlier. Cholesterol depletion, rescue, and overloading of cells were performed by methods adapted from published protocols (Hortsch et al., 2010; Christian et al., 1997). Further details, antibodies used, and procedures for immunohistochemistry of *Drosophila* tissues, ubiquitination assays, and coimmunoprecipitation are provided in the Extended Experimental Procedures.

### Luciferase Reporter Assay

S2 cells were grown in 12-well dishes and transfected with pMT plasmids, NRE:Firefly (a gift from S. Bray), and Actin:Renilla (a gift from G. Merdes). Luciferase activity was assayed with Dual-Glo Luciferase (Promega) 24 hr after induction of expression, quantified by luminometer (Berthold), and Firefly/Renilla ratio calculated. For RNAi experiments, 1 day after transfection, cells were serum starved for 1 hr and reseeded in 96-well plates with 1 μg double-stranded RNA per well for a further 4 hr before replenishing serum back to 10%. Cells were cultured for a further 2 days before adding CuSO_4_. Signaling was normalized to relevant control as indicated. Further details of RNAi and inhibitor treatments used are provided in the Extended Experimental Procedures.

### Statistical Methods

Quantified data are expressed in figures as means ± SEM. Statistical significance was determined as indicated in the figure legends utilizing SPSS software (SPSS Inc.) or GraphPad (GraphPad Software). Data for luciferase and endocytic localization experiments are displayed as mean or mean percent ± SEM from at least three experimental repeats, and statistical significance was determined by Student t test. For protein localization assays, a minimum of 60 cells or vesicles were scored for each experimental repeat. For scoring of *Drosophila* phenotypes, data are expressed as percent, and statistical significance was tested using Fisher’s exact test.

### Mathematical Modeling

A description of the mathematical model and its analysis are provided in Data File S1.

Extended Experimental Procedures ***Drosophila* Stocks**

*Su(dx)*^*-*^ (refers to heteroallelic null combination of Su(dx)^32^/Su(dx)^56^), *Su(dx)*^*sp*^, (Fostier et al., 1998; Cornell et al., 1999), *dx*^*-*^ (refers to null *dx*^*152*^ allele; Fuwa et al., 2006), *car*^*1*^ (Bloomington Stock Center, Indiana). UAS lines were UAS-Dx (Gift from K. Matsuno), UAS-Su(dx); UAS-Su(dx)ΔHECT (Cornell et al., 1999). UAS-Trpml (Venkatachalam et al., 2013), UAS-Rab7QL (Entchev et al., 2000), UAS:GPI-GFP (Greco et al., 2001). Gal4 driver lines were dpp-Gal4 (Staehling-Hampton et al., 1994), ptc-Gal4 (Speicher et al., 1994), mat-αtubGal4 (Hunter and Wieschaus, 2000). The Notch reporter element (NRE)-GFP line was as reported previously (Saj et al., 2010). The Su(dx) genomic rescue (GR) line was created by standard embryo injection technique using pCasper vector containing a genomic subclone including the *Su(dx)* gene and approximately 1.5kb of upstream regulatory region. *Su(dx)*^*GR*^ insert is localized on chromosome 2. *OregonR* was used as wild-type.

**RNAi**

RNAi used for luciferase and endocytosis assays were provided by the Sheffield University RNAi facility and based on the Heidelberg 2 BKN library (Horn et al., 2010): Mastermind (BKN30687), Dynamin (BKN21495), Rab5 (BKN22991), Rab7 (BKN28849), Dor (BKN22242), Kuz (BKN23747), GlcT-1 (BKN27304), Egghead (BKN27178) and β4GalNAcTB (BKN25023), Ser (BKN28416), CHC (BKN20463), Synaptojanin (BKN60321).

**Expression Vectors**

pMT-N was derived from subcloning of Notch cDNA from pUAST-Notch-YFP (gift of K. Matsuno), removing the C-terminal YFP tag to generate an authentic 3′ end. pMT-N^D505A^ was sub-cloned from pCasper HS-N^D505A^ (Whiteman et al., 2013) while pMT-N^R2027A^ was generated by site-directed mutagenesis, pMT-NEXT and pMT-NICD were generated from pMT-N. pMT-Dx and pMT-Venus-Dx, were generous gifts from K. Matsuno, pMT-Ser was as described previously (Whiteman et al., 2013). For Su(dx) expression we used pMT-HA-Su(dx) and pMT-HA-Su(dx)-V5 (Flasza et al., 2006). Other vectors were pUAST-EYFP-Rab7 and pUAST-EYFP-Rab5 (gifts from M.P. Scott), pUAST-GPI-GFP (gift from S. Eaton) and pMT-GAL4 (*Drosophila* Genome Research Center). The pMT-Rab5DN and pMT-Rab7DN were constructed by subcloning the respective coding sequences into pMT from pUAST-EYFP-Rab5DN and pUAST-EYFP-Rab7DN (gifts from M.P. Scott). The pMT-RFP-Rab5 and pMT-RFP-Rab7 constructs were provided by B. Rowshanravan and D. Hughes.

**Immunohistochemistry**

Antibodies were mouse anti-Notch extracellular domain (Diederich et al., 1994, C458.2H, DSHB, ascites 1:200), anti-Eve (2B8, DHSB, supernatant 1:30), Rabbit anti-GFP (Immunokontact, 1:1000) anti-HRP-FITC (Jackson Labs, 1:200). Rabbit anti-V5 (Bethyl laboratories, 1:1000), Rabbit monoclonal anti-HA (Cell Signaling, 1:200,), anti-Peanut (DSHB, 1:5000). S2 Cells were fixed in 4% formaldehyde (Polysciences) for 30 min, rinsed in PBS, permeabilised in 0.2% Triton X-100 /PBS, and blocked 1 hr in 3% skimmed milk/PBS, then incubated with primary antibody in blocking solution for 2 hr, washed in PBS before 1hr incubation with secondary antibody. Cell preps were washed in PBS and mounted in Vectashield with DAPI (Vector labs).

For staining of *Drosophila* tissues, the procedures for in situ hybridization and immunostaining of 3rd instar imaginal discs were as previously described (Cornell et al., 1999; Wilkin et al., 2004). For embryo CNS staining, stage 15/16 embryos were flat prepped and fixed for 1 hr in 4% formaldehyde, washed in PBS-Triton X-100 (0.1%), permeablised overnight at 4°C in PBS-Triton X-100 (0.3%), 4% normal donkey serum, prior to immunostaining, using PBS-TritonX (0.1%) for all washes. Tissues were mounted in Vectashield with DAPI (Vector labs).

Images captured using Volocity (Perkin Elmer) with an Orca-ER digital camera (Hamamatsu) mounted on a M2 fluorescent microscope (Zeiss). Deconvolution of 0.5 μm optical sections was performed with 3 nearest neighbors using Openlab (Improvision), or by iterative deconvolution (Volocity) and processed in Photoshop (Adobe).

**Ubiquitination Assay**

S2 cells were transfected with pMT-N-GFP, pMT-Flag-Ubi (gift from S Bray), and pMT-HA-Su(dx) or pMT-HA-Su(dx)-V5 at 25°C. After 48hr, 1mM CuSO4 was added to induce protein expression, followed by incubation at 18°C for 24hr. The S2 cells were supplemented with 50 μM MG132 (Enzo Life Sciences), 200 μM chloroquine (Sigma), and 10mM NH_4_Cl at 18°C for 1hr, then either retained at 18°C or transferred to 25°C or 29°C for 15 min. The cells were lysed in lysis buffer (50mM Tris-HCl, pH7.5, 125mM NaCl, 5% glycerol, 0.5% NP-40, 1.5mM MgCl_2_, 1mM DTT, 1mM EGTA, 1mM N-ethyl-maleimide, 10 μM MG132 and complete protease inhibitor (Roche), and pulled-down with GFP-Trap (ChromoTek).

**Coimmunoprecipitation**

For co-immunoprecipitation experiments S2 cells were grown in 6-well dishes and transfected with pMT-N constructs and pMT-Venus-Dx plasmids. CuSO_4_ was added after 24 hr to induce expression and after a further 24 hr cells were homogenized in lysis buffer (50 mM Tris-HCl, pH 8.0, 150 mM NaCl, 1% Triton X-100, 1mM CaCl_2_ and protease inhibitor cocktail (EDTA-free Complete; Roche)), and cleared by centrifugation. The lysate was incubated with 10μl GFP-TRAP agarose (Chromotek) for 1 hr at 4°C, and washed 4 times in lysis buffer. Bound proteins were eluted with sample buffer.

**Western Blotting**

Protein samples were run on 3%–8% Nupage Gels (Invitrogen). For Western blots we used mouse anti-NICD (1:5000, Fehon et al., 1990; C17.9C6, DSHB) or rabbit anti-GFP (1:20000; ImmunoKontact), rabbit anti-Rab5 (Abcam, 1:3000) or rabbit anti-Dor (1:5000). Flag-Ubiquitin was detected by mouse M2 anti-Flag (Sigma, 1:10000), HA-Su(dx) by rabbit anti-HA (Cell signaling, 1:10000). Images of Western blots were quantified by ImageJ (NIH) and normalized to staining by mouse anti-Peanut (DHSB, 4C9H4 1:5000), used as a loading control.

**Luciferase Assay**

S2 cells in 12-well dishes were transfected with pMT plasmids, NRE:Firefly (gift from S. Bray) and Actin:Renilla (gift from G. Merdes). For ligand-independent signaling after 1 day cells were re-suspended and seeded into white 96-well plates (Nunc #136101), CuSO_4_ was added after a further 24 hr. For ligand-induced signaling, 2 days after transfection CuSO_4_ was added directly to the 12-well dish before re-seeding cells into white 96-well plates on top of fixed (4% formaldehyde, 20-30min, then washed 2X in PBS and 2X in Schneider’s medium/10% FBS): Dl expressing S2 cells (S2-Mt-Dl; DGRC); cells transfected with pMT-Ser; or control S2 cells. For RNAi experiments, 1 day after transfection cells were serum-starved for 1 hr, re-seeded in 96 well plates with 1μg dsRNA per well for a further 4 hr before replenishing serum back to 10%. Cells were cultured for a further 2 days before adding CuSO_4_. Either GFP (control) or the test dsRNAs were used. For temperature-shift experiments, following CuSO_4_ addition, cells were shifted to the indicated temperatures for the remainder of the assay. For inhibitor experiments, BB94 (10 μM) or DAPT (10 μM) (Calbiochem), or vehicle (DMSO) were added at the same time as CuSO_4_. For all experiments 24hrs after induction of expression, luciferase activity was assayed with Dual-Glo Luciferase (Promega), quantified by luminometer (Berthold) and Firefly/Renilla ratio calculated for triplicate samples. Experiments repeated a minimum of three times. Signaling was normalized to relevant control as indicated.

**Cholesterol Depletion and Overloading**

The procedure to deplete cholesterol from cells was adapted from Hortsch et al., 2010, S2 cells were treated with 1% methyl-β-cyclodextrin (mβCD) (Sigma) in Schneider’s culture medium supplemented with Penicillin-Streptomycin and charcoal stripped FBS (Sera Laboratories) for 1 hr before and during uptake assay. For luciferase assays S2 cells were treated as above with 1% mβCD at the same time as adding CuSO_4_, 24hrs later luciferase activity was measured. For cholesterol-rescue or overloading experiments, 1% mβCD saturated with cholesterol was prepared in Schneider’s medium as described previously (Christian et al., 1997). For cholesterol rescue experiments a 1 hr incubation in a 10-fold dilution of this mixture in Schneider’s was used subsequent to the cholesterol depletion procedure. To overload S2 cells with cholesterol, undiluted mβCD-cholesterol containing Schneider’s medium was used. Treated cells were transferred to normal Schneider’s medium just prior to Notch endocytosis assay.

**Quantitative PCR Assay**

Total RNA was isolated from S2 cells (approximately 0.5 × 10^5^) with Tri reagent (Sigma-Aldrich). 100ng RNA was analyzed by using SensiFAST SYBR Lo-ROX One-Step Kit (Bioline) in StepOnePlus System (Life Technologies). The primers used for detection were: Ser: CATAACAACCTGTAGCGCGC, TCGCCGAATCCTTGTCGAAA; Shi: ATTCGCAAGGGTCACATGGT, ACCATCCAACGGCAGCATAA; Rab5: CATCGAACTCTACGACGCGA, CCTGGGTCAAGGAACTGCAT; Rab7: AGGGCATCAACGTGGAGATG, CGATTGTTTTGCGAGCCCAA; Mam: TATCAGCACAGCTTCTGGGAC, ACGCGGAGAGGTGTTAGGA; Dor: ACATGAAGCTGGCCAAGGAA, TGCGTAAGAGATCGCACTCC; Kuz: GAAGGCATTGCTGACCACGA, CGTCGAACTTTGTGTTGCGG; Notch: GCAAGTGCCCCAAAGGTTTC, CAGGTGTAGTCCGAGATGCC; CHC: TGACATGAACGATGCCACCA, TGCAACGTCTTTTGCGCTTT; Synj: AACTAATGGATGGTGCGCGA, GGACATCAATGGCTTCCTGC; GlcT1: CTTCGCGGCATTCTTCATGG, GCTTGCAGGACTTCTTGTGC; Egh: CGTTTTGGCATGGAAAACATGAA, GTGAACTTGGACGTGGTTGC; β4galNACTB: AACAGGGCGATGCTCTTCAA, CGAATTCAGTGGCAGGAGGT; RP49RT: AGTATCTGATGCCCAACATCG, TTCCGACCAGGTTACAAGAAC.

## Author Contributions

H.S., S.A.W., M.B.W., and M.B. designed and interpreted experiments and cowrote the manuscript with input from B.T. and N.A.M.M. H.S. performed and analyzed protein localization experiments, S.A.W. performed and analyzed cell signaling assays, and M.B.W. performed and analyzed in vivo experiments. Mathematical modeling was performed and analyzed by B.T. and N.A.M.M.

## Supplemental Information

Data S1. Mathematical Modeling, Related to Figures 5 and 6

Document S1. Article plus Supplemental Information
