# Supplementary material for: Compensatory Flux Changes within an Endocytic Trafficking Network Maintain Thermal Robustness of Notch Signaling
Source: Cell. 2014 May 22;157(5):1160–74. doi: 10.1016/j.cell.2014.03.050 (PMC4032575; doi:10.1016/j.cell.2014.03.050)
Supplement: Data S1. Mathematical Modeling, Related to Figures 5 and 6 [file mmc1.pdf]

Data File S1. Mathematical Modeling, Related to Figures 5 and 6.

We developed a computational model of the Notch signaling network, described by a set of ordinary differential equations. The model comprises three experimentally determined distinct routes to Notch activation: a) simplified canonical pathway (ligand-induced signaling), b) Deltex-dependent, late endosome-dependent, cholesterol-independent signaling pathway, and c) early endosome-dependent, cholesterol-dependent but late endosome-independent pathway (Data File S1 Figure 1).

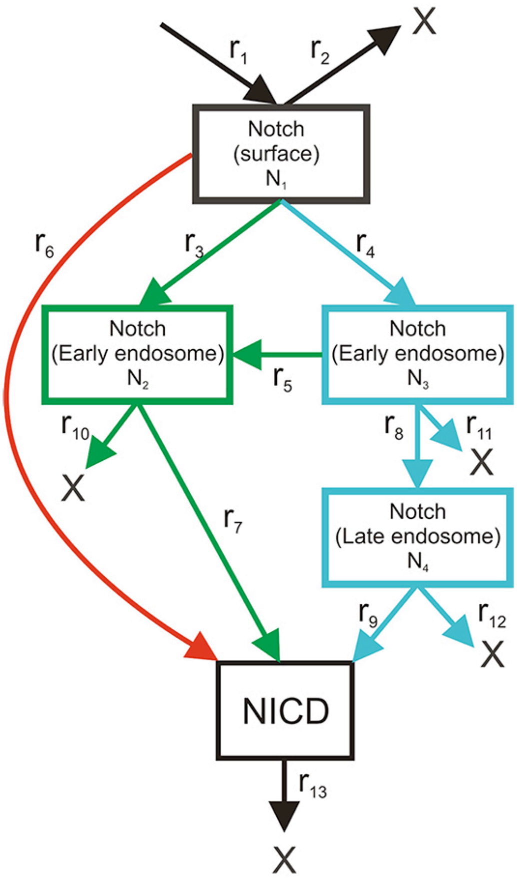

Data File S1 Figure 1. Network Diagram Implemented in Modeling Studies. Green colour represents cholesterol-dependent pathway, blue represents Dx-dependent, cholesterol-independent pathway, red represents ligand-dependent pathway. X means that Notch is degraded, while r_1-r_{13} denote rates of particular steps.

Equations for the network model of Notch signaling are given below (equations 1 - 18). As there was no quantitative information about the kinetics of the reactions used in our model, all rate equations follow mass action kinetics (Equations 1 - 13), with kinetic constants k_i and c_i, apart from (8) where a saturating term is included for the early to late endosome maturation step.

Rate equations were:

$$r_1 = k_1 \quad (1)$$

Decribes the rate of synthesis of Notch which was assumed to be constant as the simplest option requiring least parameter fitting.

$$r_2 = k_2[N_1] \quad (2)$$

This term assumes there are additional routes that can remove the receptor without signal activation beyond those routes directly under study in this model. This term allows a steady state of $[N_1]$ to be achieved without excessive accumulation when Su(dx) and Dx pathways are removed.

$$r_3 = (k_3[Su(dx)] + c_3)[N_1] \quad (3)$$

$$r_4 = (k_4[Dx] + c_4)[N_1] \quad (4)$$

The rates r_3 and r_4 represent endocytosis of Notch to the early endosome through respectively GPI-protein positive and negative vesicular trafficking. The constants c_3 and c_4 were included to represent a background Notch endocytosis observed in the absence of Su(dx) and Dx expression.

$$r_5 = k_5[Su(dx)][N_3] \tag{5}$$

Describes transfer of Notch between GPI-protein negative to positive compartments of endosomal vesicles observed when Su(dx) and Dx are coexpressed. In this case we have not assumed a background flux in the absence of Su(dx). We have no evidence for this experimentally as the proportion of Notch in GPI-protein positive compartments does not increase during a time course of Notch endocytic uptake when Dx is expressed alone (Figure. 3N main text).

$$r_6 = k_6[Ligand][N_1] \tag{6}$$

A simplified term accounting for Notch activation by ligand, there is no constant term because entry into this activation pathway is entirely ligand-dependent. This route to activation is made separate to r_7 and r_9 routes based on experimental evidence that distinguishes different mechanisms as described in the main text of the paper.

$$r_7 = k_7[N_2] \tag{7}$$

This term represents Notch activtion in the GPI-protein positive endosomal pathway which is independent of late endosomal trafficking.

$$r_8 = k_8[N_3] + \frac{c_{8a}[N_3]}{(c_{8b} + [N_3])} \tag{8}$$

This term accounts for transfer of Notch from the early endosome through the late endosome to the lysosome. The saturation term, defined by $c_{8a}[N_3]/(c_{8b} + [N_3])$, was included to take account of observations that Dx-induced signaling in the *Drosophila* wing is strongly potentiated by increasing late endosomal trafficking by expression of activated Rab7. We infer that in normal conditions the process of endosomal maturation and late endosomal fusion to the lysosome becomes rate limiting. The linear component $k_8[N_3]$ of this term was not essential to the main features and predictions of the model but was included as high N_3 concentrations resulting from

loss of Su(dx) would otherwise not lead to further increased Notch signaling when Dx was strongly expressed, as is observed in vivo. Experimentally, co-overexpression of Dx with Notch increases Notch signaling above that resulting from Dx expression alone, (data not shown) suggesting that increased Notch levels increase capacity for this endocytic activation route.

$$r_9 = (k_9[Dx] + c_9)[N_4] \tag{9}$$

Represents the Notch activation step which occurs on the lysosomal limiting membrane. The Dx term reflects a second role for Dx in promoting Notch retention on the limiting membrane, preventing Notch being sequestered into the internal luminal compartments. The c_9 term assumes a low level of Notch retention on the limiting membrane in the absence of Dx.

$$r_{10} = (k_{10}[Su(dx)] + c_{10})[N_2] \tag{10}$$

Describes the transfer of Notch from the GPI-protein positive endosomal surface into internal luminal compartments, sequestering it from activation en route to a degradative step. Su(dx) promotes this transfer through a HECT domain dependent activity. The constant c_{10} represents Su(dx)-independent transfer. A low background of transfer can be inferred from the dominant negative activity of the HECT domain inactive Su(dx)V5 construct which prevents a basal rate of transfer of Notch from the endosomal limiting membrane into the internal luminal compartments (Figure 2N,O, main text).

$$r_{11} = k_{11}[N_3] \tag{11}$$

$$r_{12} = k_{12}[N_4] \tag{12}$$

The r_{11} and r_{12} terms account for removal of Notch from access to lysosomal activation by transfer into the multivesicular body in the GPI-protein negative route which can occur at different steps of endosomal maturation. These down regulatory steps can be inferred by the

strong Notch activation that occurs when ESCRT I,II, III components are removed as described in published work (see Fortini and Bilder 2009).

$$r_{13} = k_{13}[NICD]. \tag{13}$$

This term accounts for post-activation degradation of NICD.

Amounts of Notch signaling molecules at different stages of signaling pathways $[N_i]$ are variables of the model. While $[N_1]$ represents the concentration of Notch on the cell membrane, $[N_2]$ to $[N_4]$ represent concentration of Notch in internal cellular compartments (early or late endosomes). $[NICD]$ is the final amount of the signaling molecule that is transported to the cell nucleus. Reaction rate constants k_i and c_i, as well as [Dx], [Su(dx)] and [Ligand] are parameters of the model.

Ordinary differential equations describing states of $[N_i]$ and $[NICD]$ (arbitary units) were:

$$\frac{d[N_1]}{dt} = r_1 - r_2 - r_3 - r_4 - r_6 \tag{14}$$

$$\frac{d[N_2]}{dt} = r_3 + r_5 - r_7 - r_{10} \tag{15}$$

$$\frac{d[N_3]}{dt} = r_4 - r_5 - r_8 - r_{11} \tag{16}$$

$$\frac{d[N_4]}{dt} = r_8 - r_9 - r_{12} \tag{17}$$

$$\frac{d[NICD]}{dt} = r_6 + r_7 + r_9 - r_{13}. \tag{18}$$

Steady state was found by solving

$$\frac{d[N_i]}{dt} = 0 \qquad (19)$$

$$\frac{d[NICD]}{dt} = 0 \qquad (20)$$

using Wolfram Mathematica 7. The solution of the system (available on request) was calculated for a given set of parameters to determine the equilibrium distribution of fluxes through the pathways represented in Data File S1 Figure 1. Using equations for steady state, for a given set of k_i and c_i parameters we calculated (in Matlab R2010a) total [NICD] for a range of [Dx] and [Su(dx)] concentrations from 0 (gene knock out) to 3 (3 fold overexpression).

Parameter setting and modeling of Notch signaling in *Drosophila* wing at 29ºC

Using this model we wished to identify a set of parameters which was capable of reproducing changes to Notch signaling observed after loss of function of Su(dx) or Dx components. Wild type [Dx], [Su(dx)] and [Ligand] were set to 1 and values of [Dx] and [Su(dx)] were then altered to model mutant phenotypes. We did not have a sufficiently quantitative objective function to allow automated search of parameter space. Instead parameters were initially set to 1, and then manually optimised to identify a parameter set which fit the following qualitative data from experimental observations made in fly wings at 29ºC (Data File S1 Table 1).

- In double null mutant (*dx, Su(dx)* mutant), no mutant phenotype is observed, Notch signaling is approximately at the wild type level.
- In the absence of Deltex (*dx* mutant), wild type level of Su(dx) leads to lowered Notch signaling.

- In the absence of Suppressor of Deltex (*Su(dx)* mutant), Notch signaling increases with increasing [Dx].

A set of parameters which fit these criteria are displayed in Data File S1 Table 1.

Data File S1 Table 1: Values of Kinetic Parameters in the Wing at 29ºC

k_1	1.4	k_{11}	1
k_2	10	k_{12}	70
k_3	240	k_{13}	0.06
k_4	420	c_3	320
k_5	100	c_4	350
k_6	500	c_{8a}	0.57
k_7	15	c_{8b}	10^{-6}
k_8	1.2	c_9	20
k_9	108	c_{10}	50
k_{10}	250		

A feature of the model which affected whether the simulated double *dx; Su(dx)* mutant was phenotypically equivalent to wild type was k_9 versus k_{12} ratio. By altering the rate of a single step - parameter k_9, simulations of Notch signaling resulted in different predicted outcomes of simultaneously removing both Su(dx) and Dx (Figure 6A,B main text compared to Figure 5B). Five fold reduction of k_9 led to stronger Notch signaling in the double mutant combination compared to when Su(dx) was removed alone. Thus Dx and Su(dx) cooperate to limit Notch activity. A five fold increase of the same parameter led to simulations of Notch signaling whereby the *Su(dx)* mutation failed to completely suppress loss of function *dx* mutant phenotypes. Phenotypes were predicted to be temperature sensitive as depicted. Similar outcomes were found by instead increasing k_{12} (data not shown).

Modeling of temperature sensitivity of Notch signaling

To simulate temperature sensitivity of the Notch signaling pathway, we assumed from experimental observations that only kinetic parameters k_3, k_5, k_6 and k_{10} are temperature-dependent. The set of parameters found to reproduce experimental observations of wing phenotypes at 29ºC was used as a starting point for temperature dependence simulations. Starting with the 29ºC parameter set, temperature sensitive parameters were expected to decrease linearly with decreasing temperature, until they reached value 0 (Data File S1 Figure 2). Parameter k_{10} was allowed to reach a negative value (-20) at low temperatures, to reflect a dominant negative effect of HECT inactive Su(dx) on the background Notch transfer from the endosomal limiting membrane into the multivesicular body. Note the latter value of k_{10} reduces the rate r_{10} by impacting on the basal Su(dx)-independent flux, but does not make the overall value of the r_{10} flux negative. The simulations predicted an enhancement of the *dx* mutant phenotype by removal of *Su(dx)* at low temperatures and a reversal of temperature sensitivity of the *dx* mutant phenotype, i.e. below a critical temperature the *dx* mutant phenotypes were expected to worsen as temperatures were further lowered. Predictions were experimentally verified as described in main text. The simulated temperature at which the cooperative effects of *Su(dx)* and *dx* mutants emerge depends on the parameter values chosen at 18ºC for k_3, k_5, k_6 and k_{10}. The parameter values utilised to simulate temperature changes displayed in Figure 5I in the main text are given in Data File S1 Table 2 and reflect the temperatures at which these phenotypic outcomes were observed in vivo.

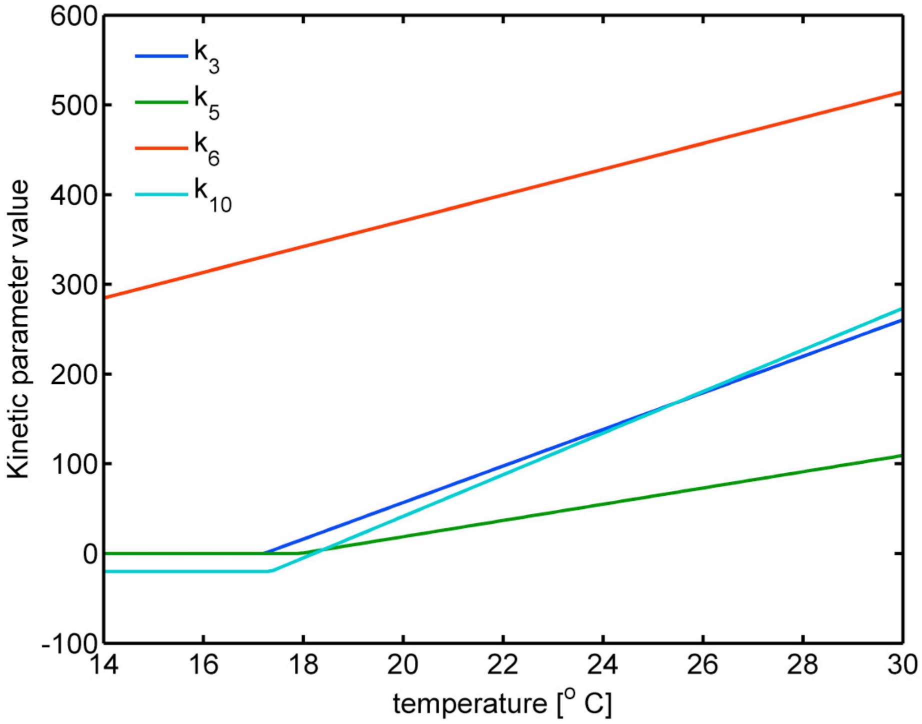

Data File S1 Figure 2. Temperature Sensitivity of Kinetic Parameters. Values of kinetic parameters at 18ºC and 29ºC were fitted to reproduce experimental observations, changing linearly, and extended to lower temperatures, flattening when a value 0 is reached. Parameter k_{10} was allowed to reach a negative value (-20) at low temperatures to reflect experimental observations.

Data File S1 Table 2. Temperature Dependent Parameters

Parameter	Value at 18°C	Value at 29°C
k_3	16	240
k_5	0.5	100
k_6	342	500
k_{10}	-5	250

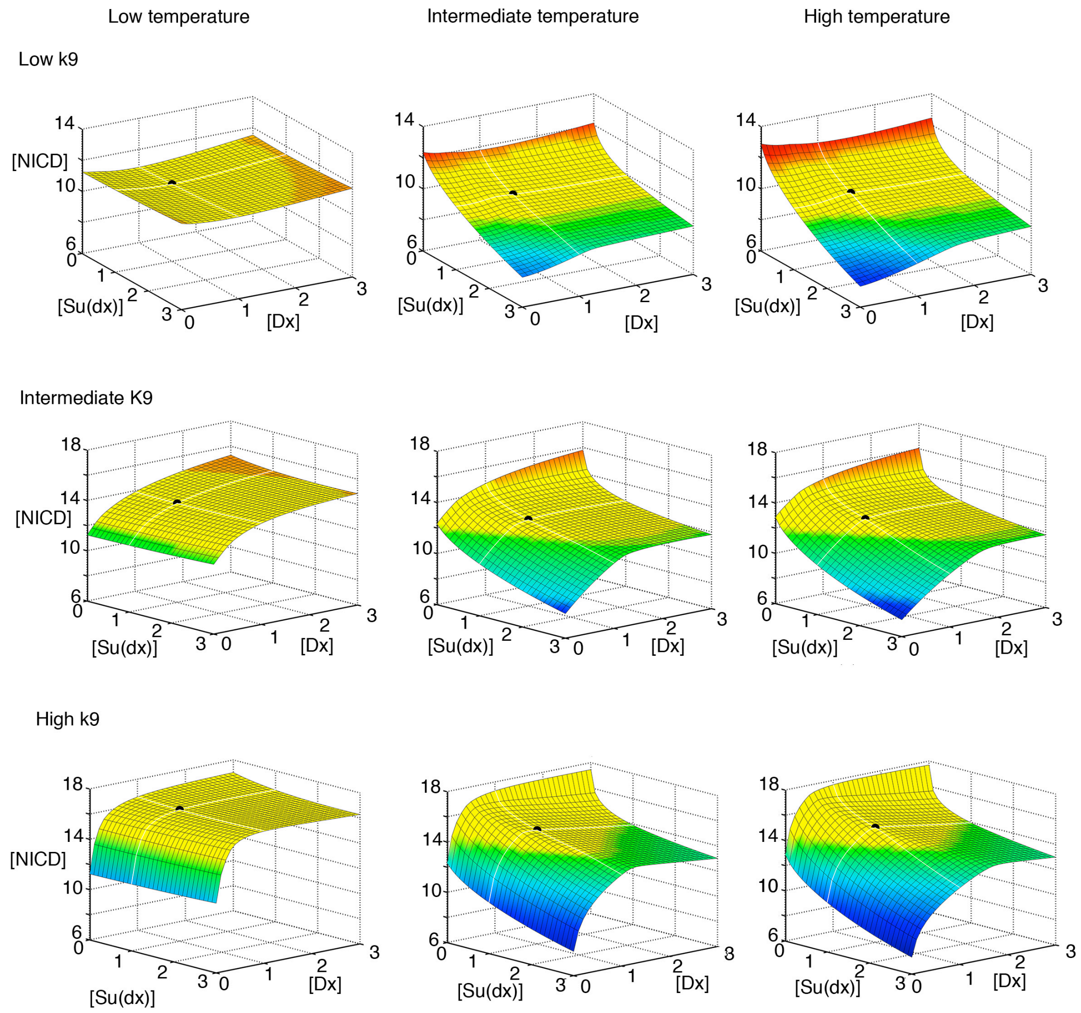

Data File S1 Figure 3. Dependency of [NICD] on [Su(dx)], [Dx] and Temperature For Low, Intermediate and High Values of k_9. At each temperature all other parameters are the same. In each case the black dot indicates wild type levels of [Su(dx)] and [Dx]. Low, intermediate and high temperature correspond to 18ºC, 25ºC and 29ºC. The values of k_9 range between between 5 fold lower and 5 fold greater than intermediate value provided in Data File S1 Table 1. Yellow shading corresponds to expected WT, orange to red represents increased expectation of Notch gain of function phenotypes, and green to blue represents increased expectation of loss of Notch function phenotypes, thresholding on graphs for low intermediate and high temperatures was based on in vivo analyses of respectively leg, wing and embryo midline phenotypes.

Temperature compensation mechanism.

Three main fluxes contribute components of NICD generation through r_6, r_7 and r_9 depicted in (Data File S1 Figure 1). As illustrated by Data File S1 Figure 4, in WT the temperature dependent increase of r_6 (ligand stimulated) is balanced by changes to r_9 (Dx-dependent) and r_7 (sterol-dependent) components, hence maintaining a flat response to temperature of the overall signaling levels. These changes are brought about by increase in temperature sensitive parameters k_3 and k_{10} which direct more Notch through the cholesterol-dependent endocytic pathway to be degraded. This flux reduces signal production through r_6 and reduces entry of Notch by the Dx-driven endocytic route r_4. The increase in temperature-dependent parameter k_5 (post-endosomal transfer between Dx and Su(dx) trafficking routes) has little effect on temperature compensation as decreasing endocytic entry through r_4 at higher temperature reduces [N_3] and hence flow through r_5. Simulations which assume k_5 is constant with temperature do not alter temperature response of overall Notch signaling (data not shown). Presence of flux through r_5 does however contribute to the observed flat response of Notch signaling to increasing Dx expression at 29ºC by maintaining [N_3] low.

Explanation of *dx* and *Su(dx)* mutant phenotypes.

Data File S1 Figure 4 illustrates how the different fluxes to NICD generation are affected by mutations in *dx* or *Su(dx)*. In a *dx* mutant, flux through r_4 is reduced to background level resulting in decreased activation through r_9 (late endosomal/lysosome route). However the consequences of the reduction in r_9 are ameliorated by increased activation through the r_6 route which is normally reduced by Dx activity. Depending on the value of the parameter k_9 the loss of *dx* can have a positive or negative consequence on overall NICD generation (Data File S1 Figure 3) and hence the effect of *dx* loss of function can be further suppressed or enhanced by

temperature-dependent changes to the competing endocytic flux through r_7 and r_{10} (Data File S1 Figure 4). The temperature dependent nature of r_6, r_7 and r_{10} thus gives rise to the temperature-dependent phenotypes of *dx* mutants at both temperature extremes.

In *Su(dx)* mutants the endocytic flux through r_7 falls to background levels and there is a small increase of flux through r_6 (ligand-dependent activation) and endocytic entry of Notch through r_4 is increased, resulting in increased Notch activation through r_9. These effects are more evident as temperature increases. The gain of function Notch phenotype observed in *Su(dx)* mutants therefore results from small temperature-dependent increases of Notch signaling through both ligand-induced and Dx-induced Notch activation pathways.

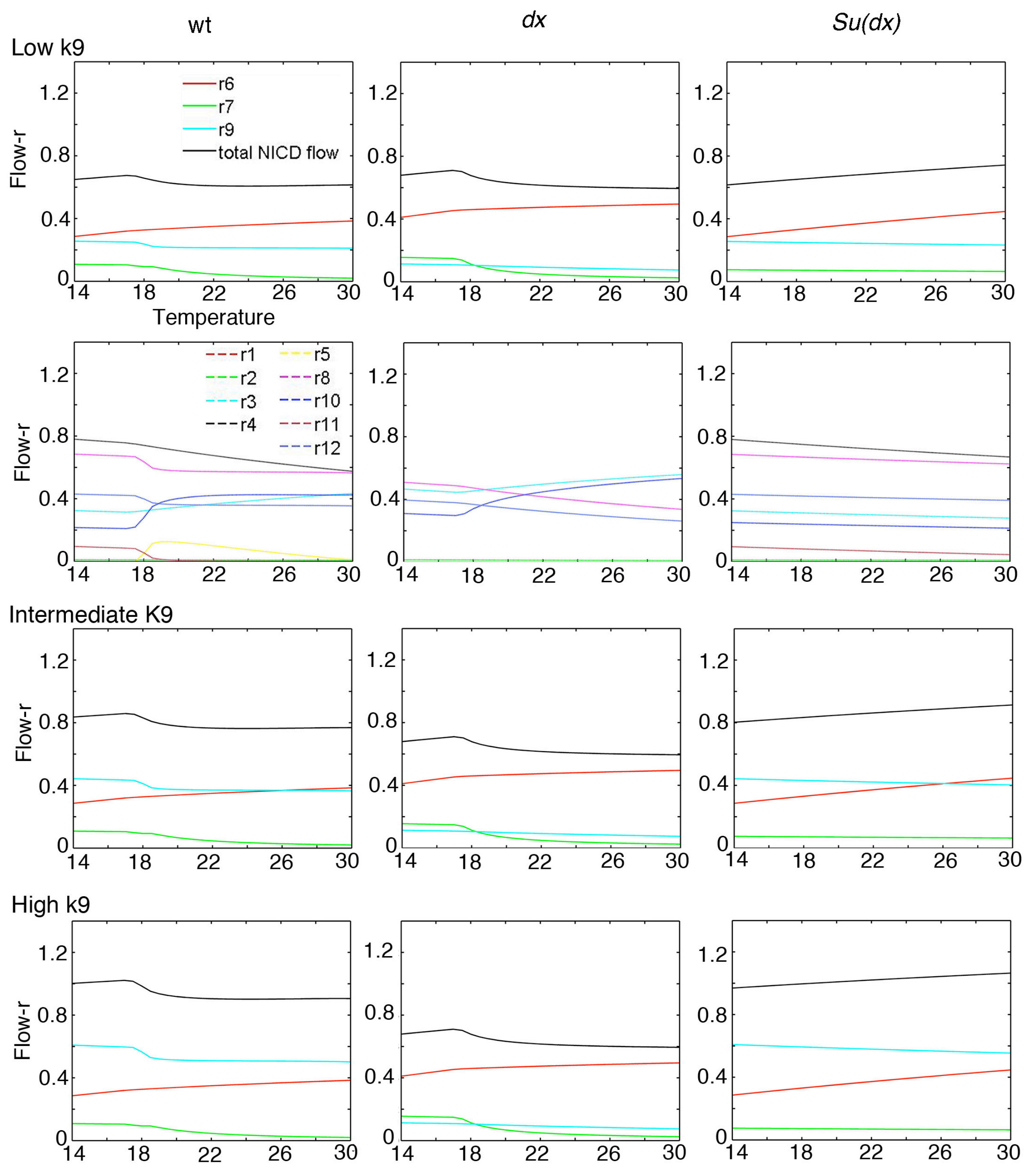

Data File S1 Figure 4. Flow Through Different Steps of the Model Over a Simulated Physiological Temperature Range. The consequences of *dx* and *Su(dx)* mutations on NICD generation are illustated for low, intermediate and high values of k_9. Changes to flow (r) through routes other than the NICD generating steps (r_6,r_7,r_9) are shown only for the low k_9 set, but similar changes are observed for all k_9 conditions.
